# Movement, demographics, and occupancy dynamics of a federally-threatened salamander: evaluating the adequacy of critical habitat

**DOI:** 10.7717/peerj.1817

**Published:** 2016-03-15

**Authors:** Nathan F. Bendik, Kira D. McEntire, Blake N. Sissel

**Affiliations:** 1 Watershed Protection Department, City of Austin, Austin, TX, United States of America; 2 Current affiliation: Warnell School of Forestry and Natural Resources, University of Georgia, Athens, GA, United States of America; 3 Current affiliation: Natural Resources, Travis County, Austin, TX, United States of America

**Keywords:** Multi-strata capture-recapture, Caudata, Dynamic occupancy models, Endangered species, *Eurycea tonkawae*, Plethodontidae

## Abstract

Critical habitat for many species is often limited to occupied localities. For rare and cryptic species, or those lacking sufficient data, occupied habitats may go unrecognized, potentially hindering species recovery. Proposed critical habitat for the aquatic Jollyville Plateau salamander (*Eurycea tonkawae*) and two sister species were delineated based on the assumption that surface habitat is restricted to springs and excludes intervening stream reaches. To test this assumption, we performed two studies to understand aspects of individual, population, and metapopulation ecology of *E. tonkawae*. First, we examined movement and population demographics using capture-recapture along a spring-influenced stream reach. We then extended our investigation of stream habitat use with a study of occupancy and habitat dynamics in multiple headwater streams. Indications of extensive stream channel use based on capture-recapture results included frequent movements of >15 m, and high juvenile abundance downstream of the spring. Initial occupancy of *E. tonkawae* was associated with shallow depths, maidenhair fern presence and low temperature variation (indicative of groundwater influence), although many occupied sites were far from known springs. Additionally, previously dry sites were three times more likely to be colonized than wet sites. Our results indicate extensive use of stream habitats, including intermittent ones, by *E. tonkawae*. These areas may be important for maintaining population connectivity or even as primary habitat patches. Restricting critical habitat to occupied sites will result in a mismatch with actual habitat use, particularly when assumptions of habitat use are untested, thus limiting the potential for recovery.

## Introduction

The term *critical habitat* refers to legally designated areas essential to the persistence and recovery of species, and endangered species law in several nations includes provisions for protection of these areas. Whether critical habitat has been successful as a tool for species recovery has been debated (Rachlinski, 1997; Hoekstra, Fagan & Bradley, 2002; Taylor, Sucking & Rachlinski, 2005; Kerkvliet & Langpap, 2007), and its shortcomings have been attributed to its practice and implementation rather than its intent (Camaclang et al., 2015). In many cases, data limitation appears to be the reason for inadequate delimitations of critical habitat (Camaclang et al., 2015). Accurate delimitation of habitat essential for the conservation of species requires basic information on both habitat associations and habitat availability (Rosenfeld & Hatfield, 2006). Conservation actions may fall short when this information is inaccurate, incomplete or disregarded (Takekawa & Beissinger, 1989; Turner et al., 2004). In practice, critical habitat is often limited only to known localities, excluding unoccupied habitats that may be important for translocations or colonization (Camaclang et al., 2015). For rare, cryptic or otherwise data-deficient species, areas that are essential to species conservation may be underrepresented by designated critical habitats, particularly if it is limited only to areas known to be occupied.

Karst fauna exemplify species that are both cryptic (in the evolutionary and ecological sense) and rare, and are often of conservation concern due to their high rates of endemicity and sensitivity to environmental perturbations (Culver et al., 2000). Among the many dozens of endemic karst fauna inhabiting the Edwards Plateau of central Texas (Reddell, 1994) is a unique group of aquatic salamanders (genus *Eurycea*), several of which have been at the center of highly publicized, political conflicts over development and the Endangered Species Act (Krausse, 1989; Haurwitz, 1993; Haurwitz, 1995; Chippindale & Price, 2005; Wermund, 2012). Many are federally listed with or without designated critical habitat (*E. tonkawae*, *E. nana*, *E. waterlooensis, E. sosorum*, *E. rathbuni*), are candidates for listing (*E. latitans*, *E. neotenes*, *E. sp.* Pedernales River Springs Salamander, *E. sp.* Comal Springs Salamander; US Fish and Wildlife Service, 2009; US Fish and Wildlife Service, 2015a) or are listed but awaiting final critical habitat determinations (*E. chisholmensis*, *E. naufragia*; US Fish and Wildlife Service, 2012). When critical habitat was recently proposed for three species (*E. chisholmensis, E. naufragia*, and *E. tonkawae*) it was limited to springs and excluded intervening areas of stream and other potential surface habitats (subsurface habitat with 300 m buffers were also proposed; US Fish and Wildlife Service, 2012). Although a few collections of epigean (surface-dwelling) populations of Texas *Eurycea* have been from stream localities (e.g., Bishop & Wright, 1937; Milstead, 1951; Bruce, 1976), the predominant view has generally been that epigean populations are restricted to the vicinity of springs (Sweet, 1982). This view may persist for several of the following reasons: (1) several high profile, single-site endemic *Eurycea* species do not occur far beyond their large, highly modified spring habitats (e.g., *E. sosorum*: Chippindale, Price & Hillis, 1993; *E. nana*: Diaz et al., 2015; *E. waterlooensis*: Hillis et al., 2001); (2) ease of collection and high abundances around springs make these areas obvious locations for ecological studies (Sweet, 1982; Bowles, Sanders & Hansen, 2006; Pierce et al., 2010; Bendik et al., 2014); or (3) physiological, morphological, or behavioral adaptations indicate the importance of groundwater-associated habitats to their evolutionary history (Stejneger, 1896; Sweet, 1978; Sweet, 1984; Chippindale et al., 2000; Bendik et al., 2013a). This habitat restriction is in contrast to most other *Eurycea* species that occupy headwater streams (in addition to seeps and springs) as aquatic larvae and paedomorphs (Petranka, 1998; Tumlison & Cline, 1997; Martin et al., 2012; Steffen et al., 2014). These habitats are also present throughout the Edwards Plateau where springs emerge to feed headwater streams, potentially creating suitable habitat for salamanders within the stream or linking habitat patches (e.g., spring outlets) as a corridor for dispersal. If intervening surface habitat between spring outlets is important, either for dispersal or as primary habitat, limited critical habitat designations and other conservation policy decisions could miss a crucial aspect of Texas *Eurycea* ecology, increase the risk of extinction for listed species and decrease their probability of recovery. Therefore, a better understanding of stream habitat and the extent to which these salamanders occupy it is necessary.

We performed two studies to examine *E. tonkawae* ecology at the individual, population and metapopulation scale to understand surface habitat use of this threatened, aquatic salamander endemic to the metropolitan area of Austin, Texas. Our first study was motivated by the surface critical habitat initially proposed for this and two similar species, which was based on the maximum distance (50 m) *E. naufragia* had been recorded to move during a single study near a spring (US Fish and Wildlife Service, 2012). Using data from repeated capture-recapture surveys during a single season, we generated estimates of superpopulation size at various distances upstream and downstream from the spring, quantified the movement of individuals between these areas, and documented the demographic structure of the population to determine whether the proposed critical habitat boundaries adequately reflect habitat used or potentially used by *E. tonkawae*.

For our second study, we extended our inquiry beyond a single population to a broad-scale study of site occupancy dynamics and habitat associations to determine the extent to which *E. tonkawae* uses headwater stream habitat. We used multi-season occupancy models to examine habitat characteristics associated with *E. tonkawae* site occupancy (MacKenzie et al., 2006) and simultaneously modeled habitat suitability dynamics in the context of wet or dry site conditions (e.g., Falke et al., 2012), which may differ among urban and rural streams. Urban streams may exhibit altered hydrologic regimes (Walsh et al., 2005), including artificially enhanced recharge from urban leakage (Sharp, 2010), potentially resulting in more wetted habitat for salamanders to use. By jointly modeling habitat and occupancy (Martin et al., 2010; Mackenzie et al., 2011), we account for imperfect detection as well as variation in stream hydrology while addressing specific questions about habitat associations and occupancy dynamics. Specifically, we ask (1) How does urbanization influence habitat availability and site occupancy? (2) What habitat characteristics are important for *E. tonkawae* occurrence? (3) How do intermittent habitat patches influence occupancy dynamics? We combine inferences from a small-scale capture-recapture effort and an expansive assessment of headwater stream occupancy and habitat dynamics to develop a better understanding of habitat use by *E. tonkawae*. We then discuss the implications of our results with regard to critical habitat.

## Methods

### Study area

Our study was conducted in the Bull Creek watershed, Travis County, Austin, Texas. The spring population case study was conducted near Lanier Spring, on the Balcones Cayonlands Preserve, a local preserve system dedicated for the protection of endangered avian and karst invertebrate fauna. Lanier Spring emerges from alluvial deposits and discharges into Bull Creek several meters from the spring opening. The presence of potential habitat upstream and downstream of the Lanier Spring confluence in the form of shallow, clear water with abundant rock cover (Bowles, Sanders & Hansen, 2006) made it an ideal site to study aspects of population biology and individual movement of * E. tonkawae* in the context of a spring-stream habitat gradient.

Headwater streams in Bull Creek are spring-fed, gaining streams (i.e., discharge increases with stream length) with flows that undulate between the surface and subsurface, particularly during dry periods. We conducted occupancy surveys within five tributaries of Bull Creek, including those whose catchments were predominantly within the Balcones Canyonlands Preserve (Mainstem/Trib. 8 & Trib. 7) and streams surrounded mostly by urban development (Trib. 4, Barrow Hollow, & Trib. 2).

### Study 1: movement and distribution around a spring

We surveyed Lanier Spring every two weeks from January through April 2013 and delimited nine 5 m survey sections around the spring outlet (four upstream, four downstream, and one at the spring outlet) every 15 m. The wetted channel boundaries determined the survey width, as each 5 m section was surveyed from bank to bank. Based on previous capture-recapture work at this site (Bendik et al., 2013b), we suspected the number of salamanders available for capture (and recapture) would be enough to detect upstream or downstream movements of individuals between sections. Detection of movement was restricted to the stream channel because this species is aquatic and overland movements are not possible. Sections were exhaustively searched for salamanders by flipping cover objects such as rocks and leaf litter. Once detected, salamanders were caught using dip nets and photographed on a standardized 0.5 cm grid. Measurements of body length were obtained from photographs (following Bendik & Gluesenkamp, 2013) using Image J (Rasband, 1997). We cropped photos to include only the head and used Wild-ID (Bolger et al., 2012) for individual identification based on pigmentation patterns. A previous study demonstrated that identification errors using this technique were lower (false rejection rate = 0.76%) compared to visible implant elastomers (VIE; false rejection rate = 1.90%) for *E. tonkawae* (Bendik et al., 2013b). Therefore, we were confident in our ability to correctly identify individuals. In addition to documenting intra-seasonal movement, we were interested in the presence and location of VIE individuals (*N* = 1, 115 marked from 2007 to 2011) from a prior study at this site. Their presence in areas beyond the spring outlet may indicate dispersal from the spring.

To compare abundances in and around the spring, we estimated superpopulation sizes for each 5 m section during the four-month sampling period using the POPAN model in program MARK 8.0 (White, 2015). The superpopulation is the abundance of all animals associated with the study system (including those within and outside of the sample site). We used model-averaged values to estimate superpopulation sizes at each section. Our model set included combinations of either time-varying or constant detection probability (*p*) and apparent survival (*ϕ*); we fixed probability of entry to vary by time (but not section) and superpopulation size by section. We also investigated the effect of body length on *p* and *ϕ*.

To calculate the probability of movement while accounting for detection error, we used a multi-state model with live-recaptures in program MARK 8.0 (White, 2015). This is an extension of the Cormack–Jolly–Seber model for calculating apparent survival and detection probability in open populations that allows for movement between strata with transition probabilities (Hestbeck, Nichols & Malecki, 1991; Brownie et al., 1993). We considered models whereby salamanders could either remain in their current position, or move to a new section. Transition probabilities estimate the rate movement between sections, an average distance of 20 m. Movement probability was calculated as twice the transition probability, since transitions in both directions indicate movement. Our model set included all combinations of time variance for apparent survival (*ϕ*), detection (*p*) and transition probabilities (*ψ*), but we did not incorporate differences among states. We also tested for the influence of body length on apparent survival, detection, and transition probability. Program U-CARE (Choquet et al., 2009) was used to estimate the $\hat {c}$ ratio, a measure of goodness-of-fit, for both the multi-state and POPAN models.

### Study 2: habitat-occupancy dynamics

Starting at the downstream confluence below the nearest known *E. tonkawae* locality, we surveyed 97 sites in 2013, and surveyed an additional 30 sites in 2014 and 2015, for a total of 127 sites (encompassing over 12 km of linear stream channel). Of those, 118 contained wetted habitat at some point during our sampling. The distribution of sites was nearly evenly split between those occurring within preserves (*n* = 57) and those in urbanized tributaries (*n* = 61). With the exception of avoidance of deep pools (>0.6 m) such as man-made impoundments, we selected sites in a systematic, random fashion. Site selection started at a random point 0–100 m from the confluence and proceeded upstream, while maintaining equal distances among sites. Because we were targeting an even distribution of sites among tributaries and urban/non-urban catchments, sites in Barrow Hollow, Trib. 2, Trib. 4 and Trib. 7 were spaced 70 m apart, while those in Mainstem/Trib. 8 were spaced 140 m apart due to the larger size of that tributary. Each site was 10 m in length (from downstream to upstream) and the wetted channel boundaries determined the width of each site. Sites were surveyed each year from 2013 through 2015; our survey seasons generally started in late March and ended in early May. Because sampling occurred once per year, we use the term “year” to refer to occupancy seasons. Two experienced observers simultaneously performed a time-limited search removing cover objects for 5 min at each site, three times per year, at a minimum interval of one week. During our surveys, we documented new spring localities where we observed water issuing from conduits within or near the stream channel and included previously documented springs (City of Austin, 2015) to compare to the distribution of occupied sites.

We also collected a suite of environmental data at each site, which was reduced to a smaller set of covariates based on our specific hypotheses of interest (see below) and an assessment of multicollinearity using eigenvector analysis. Scatter plots among candidate predictive variables and an assessment of multicollinearity are included in Supplemental Information 1. We conducted one-way ANOVAs among continuous predictor variables and site groupings (tributary or urbanization) and Chi-squared tests among categorical variables and groupings. The program R (R Core Team, 2014) was used for data preparation and preliminary statistical analyses. Significance was evaluated at *α* = 0.05.

We used a model that integrates both habitat and occupancy dynamics to fit detection/non-detection data and changes in habitat suitability (Mackenzie et al., 2011). We used a simplified version of this model, with only three possible states: suitable, but species undetected; suitable with species detected; or unsuitable and unoccupied. Because neotenic *Eurycea* are stream-dwelling, aquatic organisms, we classified sites that had flowing water during some portion of our sampling season as suitable (S) and those that were either dry during the entire period, or initially stagnant and later became dry, as unsuitable (U). In this context, “suitable” habitat refers to a minimum condition for the presence of an aquatic species, and beyond that does not imply that such habitat is necessarily of good quality. Following the notation of Mackenzie et al. (2011), the parameter *π*^[*S*]^ represents the probability of a site being suitable habitat during the first year. The probability of habitat changing state between years is represented by two parameters: ${\eta }_{t}^{[S,S]}$ represents the probability of habitat remaining suitable between year *t* and *t* + 1; ${\eta }_{t}^{[U,S]}$ is the probability of habitat transitioning from unsuitable to suitable between year *t* and *t* + 1. The probabilities of habitat to transition from suitable to unsuitable, or to remain unsuitable are 1 $-{\eta }_{t}^{[S,S]}$ and 1 $-{\eta }_{t}^{[U,S]}$, respectively. The probability the species was initially present at a site is represented by *ψ*^[*S*]^ (initial occupancy probability for unsuitable sites is fixed at zero). The probability a species colonizes a site between years *t* and *t* + 1 is described by two parameters, ${\gamma }_{t}^{[S,S]}$ and ${\gamma }_{t}^{[U,S]}$. The probability a species goes extinct at a site between years *t* and *t* + 1 is ${\varepsilon }_{t}^{[S,S]}$. By definition, when habitat becomes unsuitable, colonization does not occur (${\gamma }_{t}^{[S,U]}={\gamma }_{t}^{[U,U]}=0$) and extinction does (${\varepsilon }_{t}^{[S,U]}=1$). Detection probability of habitat state was assumed to be perfect and we also assumed that species occupancy had no bearing on habitat suitability. A primary assumption of the occupancy model we used required that both habitat suitability and occupancy remained constant within each sampling season.

With regard to habitat dynamics, we predicted that habitat suitability would be dependent upon the prior state because spring locations and other geomorphological factors that influence hydrologic periodicity are assumed to be relatively constant. We therefore compared models where habitat suitability exhibited a first-order Markovian pattern of state dependence (represented by two parameters, ${\eta }_{t}^{[S,S]}$ and ${\eta }_{t}^{[U,S]}$) to those where habitat suitability was independent of the previous state (${\eta }_{t}^{[S,S]}={\eta }_{t}^{[U,S]}$). Our first year of sampling was also the driest, so we expected that urban tributaries would have more wet sites than non-urban tributaries because of artificial groundwater recharge from irrigation and leaking treated water and wastewater infrastructure (Christian, Banner & Mack, 2011). To test for this effect, we considered models where the initial habitat state *π*^[*S*]^ was a function of the group variables tributary (TRIB) or urbanization (URB). Sites are nested within tributaries, and tributaries are nested within the urbanization classification. URB is a tributary-wide categorical variable based upon predominant land use within the catchment. Compared to those within preserves, urbanized tributaries are characterized by having markedly higher water conductivity (Supplemental Information 1 and Figure S4) and are surrounded by residential development.

We also tested the importance of TRIB and URB groupings with regard to site occupancy and colonization. The initial descriptive analysis we performed indicated both tributary and urbanization groupings could be explained by certain environmental covariates. Therefore, we modeled the effects of group and environmental explanatory variables on the parameters of interest separately.

To determine what habitat conditions were associated with site occupancy, we compared models where initial occupancy and colonization were dependent upon a combination of habitat covariates. We initially collected a suite of environmental variables and narrowed down this set to a candidate list of possible predictors based on biological hypotheses and elimination of redundant or collinear variables (see Supplemental Information 1). Because previous studies of central Texas *Eurycea* have demonstrated the importance of groundwater habitats, we were interested in variables that could be reliable predictors of groundwater influence at a given site. Seasonal variation in groundwater temperature is much less than that of surface water, so sites with lower variation in temperature within sampling seasons could indicate groundwater-influenced habitats such as groundwater springs and stream-fed “springs” (created when upstream flow sinks underground, reemerging downstream). We measured water temperature during each survey and computed temperature variation as the standard deviation of temperature (TS) within each year. Additionally, we also used the bankside presence of maidenhair ferns (MH), *Adiantum sp.*, as potential indicators of groundwater or water permanence. These plants are commonly found near springs and seeps in Texas (Brune, 1981). We predicted low water temperature variation and fern presence would be positively correlated with salamander occupancy and colonization. Site occupancy within these stream reaches may also be predicted by other aspects of their habitat. Rock cover (RC) is an important habitat characteristic that is often correlated with presence and abundance of *Eurycea* (Bowles, Sanders & Hansen, 2006; Pierce et al., 2010; Diaz et al., 2015). RC was measured as the mean of three visual estimates of the percentage of rocks available (i.e., unembedded) for cover (>8 mm) at the substrate surface. Water depth (WD) may be important for exclusion of fish predators (although we purposely avoided sampling deep pools) so we expected it to negatively affect occupancy. However, WD may also influence dry risk and temperature variation as well. WD was taken as the mean of three evenly spaced measurements at the thalweg. Sites with high calcium carbonate (CA) cementation may not be preferred habitat (Sweet, 1982), lacking enough interstitial spaces for salamanders or their prey items. CA was visually estimated as being in one of four categories: (1) no carbonate build-up; (2) low to moderate build up (but <50% area); (3) moderate to high build up (>50% area); and (4) complete covering of area with carbonate and tufa. For analyses, these categories were collapsed into low (1 or 2) and high (3 or 4); 43% of observations had no carbonate cementation and only two observations were ranked 4. We incorporated yearly differences in TS, WD and RC in our analysis, but assumed that CA and MH did not change (we used the single, maximum value recorded among all years). Because missing covariate data are not allowed in occupancy models, we used the within-tributary average for 5 missing values of WD and 1 of TS (1% of measurements for those covariates) in lieu of excluding those sites from our analysis. Twelve survey events with available occupancy data were missing most covariate values due to having a single visit (e.g., temperature variation measurements required at least two visits), and were dropped from the analysis.

To understand how habitat instability influences occupancy dynamics, we compared models where colonization was dependent on the prior habitat state (${\gamma }_{t}^{[U,S]},{\gamma }_{t}^{[S,S]}$) or was random with respect to prior habitat (${\gamma }_{t}^{[U,S]}={\gamma }_{t}^{[S,S]}$). Estimates of ${\gamma }_{t}^{[U,S]}$ are a measure of the ability to colonize a previously dry site, which may indicate migration between stable spring habitats and/or an ability to take advantage of habitats throughout the stream during wetter years. Our sampling occurred over a period of progressively wetter conditions, so we were able to determine whether dry sites were eventually colonized or not. Covariates were mirrored among *ψ* and *γ*_*t*_ to simplify the model set, and because we were interested in the set of covariates that most consistently predicted whether a site could be occupied or not. For example, if we included WD (from year 1) as a covariate on *ψ*^[*S*]^, *γ*_1_ and *γ*_2_ were also modeled with WD (corresponding to year 2 and year 3, respectively). The effect of environmental variables on colonization was assumed to be consistent regardless of the prior habitat state. Continuous covariates were standardized for analysis by subtracting the arithmetic mean from each value.

The program PRESENCE 9.7 (Hines, 2006) was used to fit models using maximum likelihood. We employed a multi-stage approach to model selection similar to those used in Dugger, Anthony & Andrews (2011), Lee & Bond (2015), and MacKenzie et al. (2012), as follows: for each stage we held fixed a general model structure while varying the structure for the focal parameter(s); then, from each model set we determined the best model using small-sample Akaike’s Information Criterion (AICc) values, keeping the best structure for the focal parameter(s) constant for each subsequent step. We estimated the effective sample size as the average value of the total number of sites and the total number of individual surveys. First, we held habitat and occupancy parameters fixed with full time and state dependencies while we modeled detection as either constant, time variant among (but not within) years (YEAR), constant among tributaries (TRIB), or as additive, YEAR + TRIB. Proceeding from the best detection structure, we then tested habitat structures (as above), keeping occupancy time and state dependent. For the third step we compared models with and without state dependency (but with time variance), including a random occupancy model, imposing the constraint *γ*_*t*_ = 1 − ε_*t*_, whereby changes in occupancy were independent of the prior state (MacKenzie et al., 2006). We then tested whether group effects TRIB or URB better explained variation in *ψ*^[*S*]^ and *γ*_*t*_ compared to the more general unstructured model. We included site-level covariates (TS, WD, RC, CA, MH) as explanatory variables in place of the group effects on *ψ*^[*S*]^ and *γ*_*t*_ to distinguish among hypotheses of habitat selection. We first determined the best structure of continuous, time-varying covariates (TS, WD, RC), to account for environmental variation in occupancy dynamics among years. Using the best combination of time-varying covariates, we then included variables MH and CA to determine if they added explanatory value to the model. We used the sum of AICc variable weights to quantify the relative importance of covariates. We model-averaged parameter estimates on the real scale and model averaged covariate coefficients (following Lukacs, Burnham & Anderson, 2010). Finally, because we used a multi-stage approach to model building, we re-tested detection and habitat model structures based on the top model to see if they were consistent with the initial decisions made in the model testing process.

## Results

### Study 1: movement and distribution around a spring

**Figure 1:**
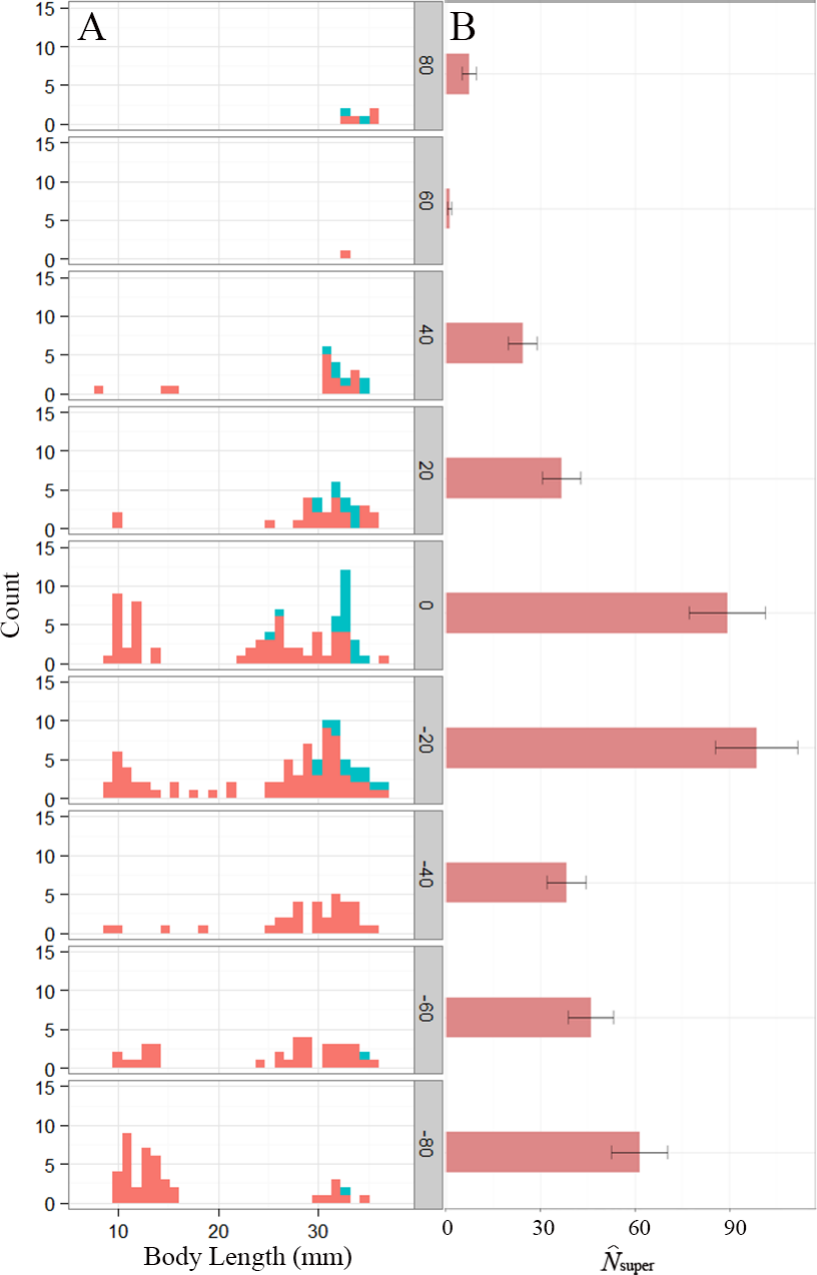
Distribution of *Eurycea tonkawae* near Lanier Spring, Bull Creek, Travis County, Texas. (A) Histogram of body length for VIE-tagged (teal) and non-tagged (red) *E. tonkawae* captured from January–April, 2013 for each section. The shaded *y*-axis represents mean distance from the spring (m) for each section. Plot includes unique individuals per section, identified by photo matching. (B) Superpopulation size estimates (± SE) for each section.

Unique individuals were recaptured in all nine survey sections. Individuals moved frequently between sections along the spring-stream gradient. Throughout the four-month period we captured 215 unique individuals (>25 mm total length) and recaptured 81 at least once. Of the recaptured animals, 21 were identified in two different sections, implying a minimum movement distance of 15 m at least once. Fourteen moved one section (an average distance of 20 m), four moved two sections (40 m), two moved three sections (60 m) and one moved four sections (80 m). We did not observe strong directionality in movement: 10 moved downstream, 11 moved upstream. We observed VIE-tagged individuals in all but two sections, including both sections 80 m from the spring (Fig. 1). Incidentally, during the 2014 occupancy surveys, we observed a single VIE-tagged individual over 500 m upstream of Lanier Spring. This individual had been last observed in 2010.

The single-state goodness-of-fit analysis indicated a significant effect of transience, which is expected given the movement between sections we observed. There was also some evidence of overdispersion in the POPAN model ($\hat {c}=1.58$), therefore adjusted QAICc values were used for model selection and model-averaging. Model-averaged estimates of superpopulation size were greatest near the spring and 20 m downstream, although the section farthest downstream had the third highest estimate (Fig. 1). Small juveniles (<25 mm total length) were most abundant downstream, with the largest proportion (32%) occurring in the most distant section (Fig. 1), possibly accounting for the high superpopulation size estimate in that section. Body length was an important covariate in our model set, contributing to 98% of the QAICc weights (including any model where body length was a covariate), although the most optimal models only included body length on either *ϕ* or *p*, not both (Table 1). Model-averaged estimates of bi-weekly survival and detection probability based on mean body length (24.8 mm) ranged from 0.44–0.66 and 0.35–0.53, respectively.

**Table 1:** Model selection results for the top five POPAN models.

Model	QAICc	ΔQAICc	*w*	*K*	−2*l*
*φ*(BL) *p*(.) *PENT*(*t*) *N*(section)	599.44	0	0.24	18	561.5
*φ*(*t*) *p*(BL) *PENT*(*t*) *N*(section)	600.22	0.77	0.16	24	548.8
*φ*(.) *p*(BL) *PENT*(*t*) *N*(section)	600.69	1.24	0.13	18	562.8
*φ*(*t* + BL)*p*(.) *PENT*(*t*) *N*(section)	600.94	1.50	0.11	24	549.6
*φ*(BL) *p*(*t*)*PENT*(*t*) *N*(section)	601.43	1.99	0.09	25	547.7

**Notes.**

*φ* apparent survival *p* detection probability PENT probability of entrance *N* superpopulation size BL body length covariate t time variation (.) constant

Shown are quasi-likelihood Akaike’s Information Criterion values corrected for small samples (QAICc) and difference from the top model (ΔQAICc), the QAICc model weight (*w*), total number of parameters (*K*) and twice the negative log-likelihood for each model adjusted for overdispersion (−2*l*).

**Figure 2:**
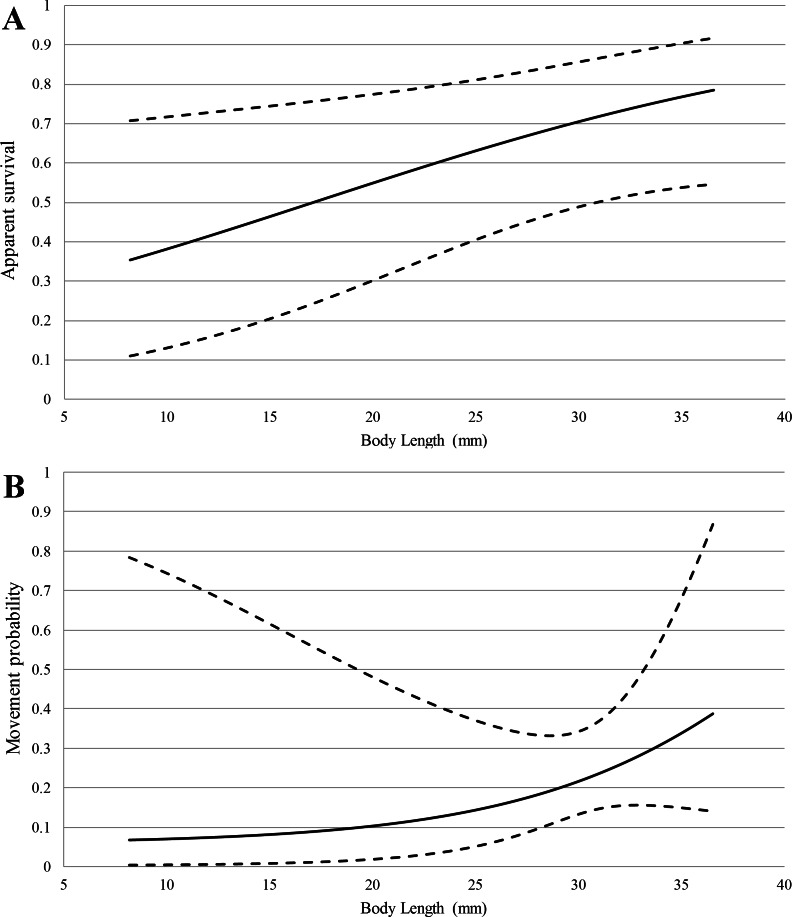
Model-averaged estimates (solid lines) of apparent survival (A) and movement probability (B) vs. body length with 95% confidence intervals (dashed lines) from multi-state capture-recapture analysis.

For the multi-state capture-recapture model, goodness-of-fit was adequate and we did not find evidence of overdispersion ($\hat {c}=0.91$). Body length was also an important predictor of both survival and movement probability (Table 2), and was positively correlated with both (Fig. 2). The sum of AICc weights for models including body length as a covariate for *φ* and *ψ* was 0.92 and 0.74, respectively. Model-averaged estimates of bi-weekly survival and detection probability based on mean body length (24.8 mm) ranged from 0.61–0.66 and 0.11–0.55, respectively. Movement probability, corrected for imperfect detection, was 0.15 (SE = 0.07) for an average length salamander. A full list of model selection results and model-averaged parameter estimates for both capture-recapture analyses are provided in Supplemental Information 2.

**Table 2:** Model selection results for the top five multi-state models.

Model	AICc	ΔAICc	*w*	*K*	−2*l*
*φ*(BL) *p*(*t*) *ψ*(BL)	767.23	0	0.41	11	744.5
*φ*(BL) *p*(*t* + BL) *ψ*(BL)	768.77	1.54	0.19	12	743.9
*φ*(BL) *p*(*t*) *ψ*(.)	769.30	2.07	0.15	10	748.7
*φ*(BL) *p*(*t* + BL) *ψ*(.)	770.82	3.59	0.07	11	748.1
*φ*(*t* + BL) *p*(*t*) *ψ*(BL)	771.82	4.59	0.04	16	738.2

**Notes.**

*φ* apparent survival *p* detection probability *ψ* transition probability BL body length covariate *t* time variation (.) constant

Shown are Akaike’s Information Criterion values corrected for small samples (AICc) and difference from the top model (ΔAICc), the AICc model weight (*w*), total number of parameters (*K*) and twice the negative log-likelihood for each model (−2*l*).

### Study 2: habitat-occupancy dynamics

**Figure 3:**
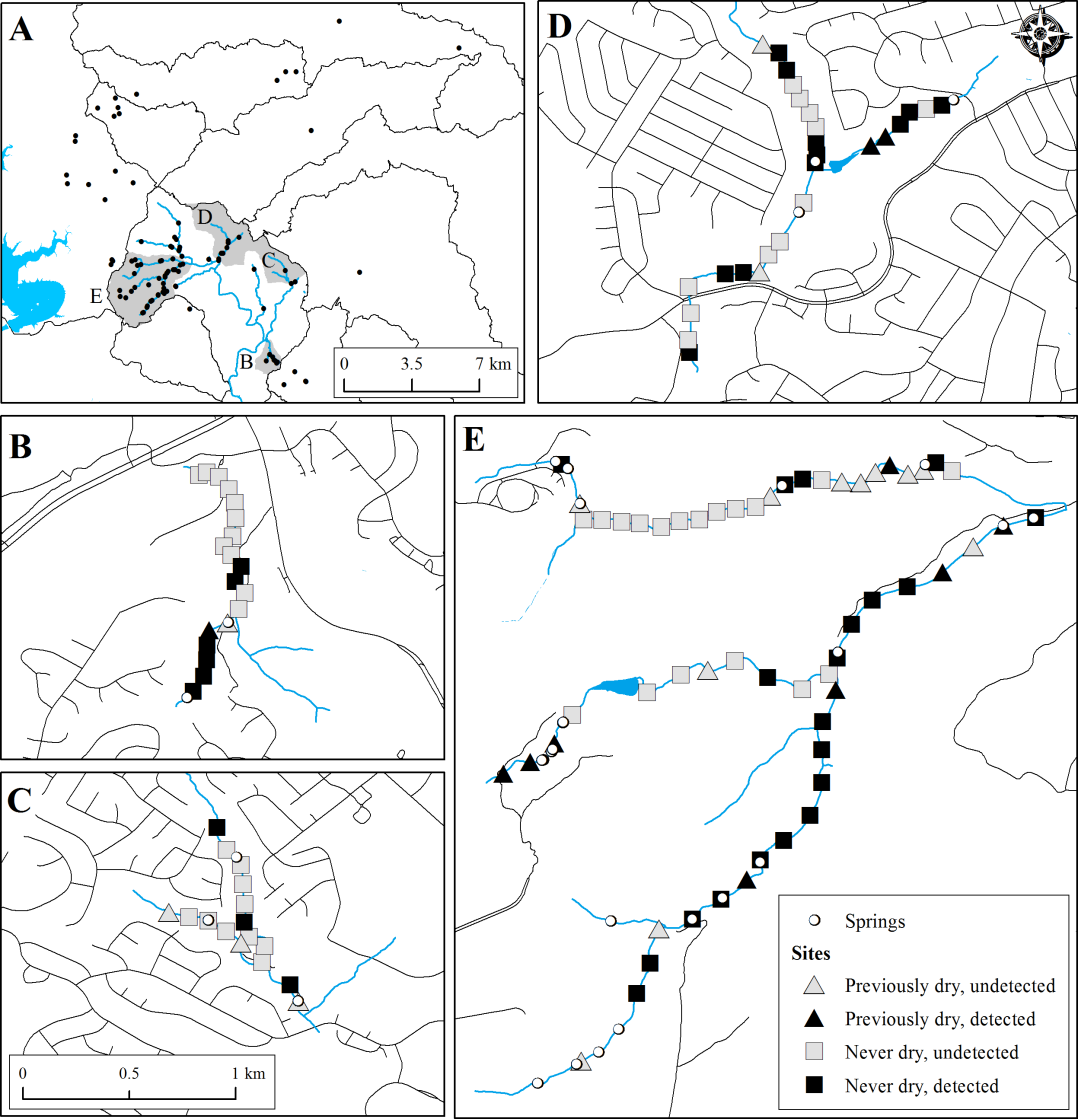
Map of *E. tonkawae* range (A) and occupancy study sites (B–E). (A) All known localities of *E. tonkawae*, watershed boundaries, and creek centerlines for major tributaries of Bull Creek. (B–E) Survey sites indicating *E. tonkawae* detection, location of known springs, and whether site was dry during any prior survey among sub-watersheds of Bull Creek. (B) Barrow Hollow. (C) Trib. 2. (D) Trib. 4. (E) Trib. 7 (top) and Mainstem/Trib. 8 (bottom). Road centerlines adjacent to tributaries are shown as an indicator of urbanization within each catchment.

We documented *E. tonkawae* nearby and well beyond previously recognized spring locations among 118 sites and five tributaries of Bull Creek (Fig. 3). The first year was the driest, and the proportion of wet sites progressively increased each year. These resulted in an overall positive trend in WD and a negative trend in TS each year (Table 3). We found significant effects of both tributary- and urbanization-based groupings for TS, RC (Table 3), MH, and CA (for results of statistical analyses, see Supplemental Information 1).

**Table 3:** Mean ±1 SD of continuous covariates among all occupancy sites.

Variable	2013	2014	2015
^a^Temperature variation (°C)	2.11 ± 1.62^b^	1.77 ± 1.14^b^^,^^c^	1.21 ± 0.685^b^^,^^c^
^a^Depth (cm)	13.3 ± 7.50	14.4 ± 7.29	15.7 ± 7.48
Rock cover (%)	44.5 ± 33.8^b^^,^^c^	35.8 ± 28.8^b^^,^^c^	35.5 ± 29.6^b^^,^^c^

**Notes.**

a Significant trend among years determined by linear regression.

b Significant difference among tributaries determined by one-way ANOVA.

c Significant difference among urbanized and non-urbanized tributaries determined by one-way ANOVA.

Based on the multi-stage modeling approach, the best model prior to including environmental covariates had a model weight of 0.94 and the following structure: tributary-dependent detection, *p*(TRIB); year- and state-dependent changes in habitat suitability (${\eta }_{t}^{[S,S]}$, ${\eta }_{t}^{[U,S]}$); initial habitat suitability, *π*^[*S*]^(URB), where sites grouped by urbanization category outperformed a tributary-based structure; Markovian site occupancy, dependent upon both the prior habitat and prior occupancy states for each year (${\gamma }_{t}^{[U,S]}$, ${\gamma }_{t}^{[S,S]}$, ${\varepsilon }_{t}^{[S,S]}$); initial occupancy, *ψ*(TRIB), where sites grouped by tributary outperformed those grouped by urbanization category (evidence ratio for *ψ* (TRIB) vs. *ψ*(URB) models = 23). Based on this model structure, we compared models with environmental covariates on initial occupancy (in place of the group structure due to redundancies; Table 3) and colonization, resulting in two top models with a combined weight of 0.99 (Table 4). Re-testing all detection- and habitat-based model structures on the top model did not alter the model hierarchy. The following results are based on model-averaged estimates from the top two models.

**Table 4:** Summary of the top five occupancy models.

Model	AICc	ΔAICc	*w*	*K*	−2*l*
*ψ*^[*S*]^ *γ*^[*S*,*U*]^ *γ*^[*S*,*S*]^ (TS_*t*_ + WD_*t*_ + MH)	753.76	0	0.51	24	703.74
*ψ*^[*S*]^ *γ*^[*S*,*U*]^ *γ*^[*S*,*S*]^ (TS_*t*_ + WD_*t*_ + CA + MH)	753.87	0.11	0.48	26	699.50
*ψ*^[*S*]^ *γ*^[*S*,*U*]^ *γ*^[*S*,*S*]^ (TS_*t*_ + WD_*t*_)	762.62	8.86	0.01	22	716.92
*ψ*^[*S*]^ *γ*^[*S*,*U*]^ *γ*^[*S*,*S*]^ (CA + TS_*t*_ + WD_*t*_)	766.14	12.38	0	24	716.12
*ψ*^[*S*]^ *γ*^[*S*,*U*]^ *γ*^[*S*,*S*]^ (RC_*t*_ + TS_*t*_ + WD_*t*_)	769.74	15.98	0	25	717.55

**Notes.**

TS standard deviation of temperature WD water depth RC rock cover CA calcium carbonate deposition MH maidenhair fern presence

All models shown included the following structure: ε^[*S*,*S*]^(*t*), *π*^[*S*]^(URB), *η*^[*S*,*S*]^(*t*), *η*^[*U*,*S*]^(*t*), *p*(TRIB). Effective sample size was assumed to be 620. Covariates were applied to all parameters represented above and their effect on colonization probabilities was assumed to be consistent regardless of prior habitat state. *t* indicates yearly variation. Shown are the Akaike’s Information Criterion values corrected for small samples (AICc) and difference from the top model (ΔAICc), the AICc model weight (*w*), total number of parameters (*K*) and twice the negative log-likelihood for each model (−2*l*).

Initial habitat suitability was higher in urbanized streams (${\hat {\pi }}^{[S]}=0.89$, SE = 0.05) compared to those in preserves (${\hat {\pi }}^{[S]}=0.63$, SE = 0.07). The probability of a site remaining wet from 2013 to 2014 was high (${\hat {\eta }}_{1}^{[S,S]}=0.96$, SE = 0.02), while the probability of a site transitioning from dry to wet during the same time period was lower (${\hat {\eta }}_{1}^{[U,S]}=0.26$, SE = 0.10). All sites were wet during the final year of sampling, resulting in both estimates of *η*_2_ at the boundary of 1.00.

The best AICc (effective sample size = 620) time-varying covariate models of initial occupancy and colonization contained covariates TS and WD (Supplemental Information 2 and Table S13), each with 100% of the relative variable weights compared to 3% relative weight for RC (calculated from the model set including all combinations of TS, WD, and RC). Consistent with our hypotheses, estimates of TS and WD coefficients had a significant negative effect on initial occupancy with 95% confidence intervals excluding zero (Table 5). Inclusion of CA and MH categorical site-level covariates improved upon the continuous covariates model, decreasing the AIC value by over 8 units. MH was positively correlated with salamander presence, while CA was negatively correlated, though not significantly (Table 5).

**Table 5:** Model-averaged coefficients (*β*), unconditional standard errors (SE) and 95% confidence intervals (CI) of intercepts and top covariates on initial occupancy (logit-scale) and colonization (logit-scale) estimating dynamic occupancy of *E. tonkawae* from 2013 to 2015 in Bull Creek, Travis County, Texas.

Initial occupancy (*ψ*)				Colonization (*γ*)			
Covariate	*β*	SE	CI	Covariate	*β*	SE	CI
Intercept	−4.30	1.49	−7.27, −1.42	Intercept[*S,S*]	−2.24	0.53	−3.28, −1.21
WD	−0.32	0.12	−0.56, −0.09	Intercept[*U,S*]	1.74	0.69	0.40, 3.09
TS	−2.88	1.10	−5.03, −0.72	TS_1_	−0.24	0.49	−1.19, 0.71
CA	−1.07	1.22	−3.47, 1.33	TS_2_	0.70	0.51	−0.30, 1.70
MH	4.07	1.52	1.09, 7.04	CA	−0.22	0.51	−1.22, 0.78
				WD_1_	0.04	0.08	−0.12, 0.20
				WD_2_	−0.04	0.06	−0.15, 0.07
				MH	−0.06	0.78	−1.59, 1.46

**Notes.**

TS standard deviation of temperature WD water depth CA calcium carbonate deposition MH maidenhair fern presence

In contrast to initial occupancy probabilities, the magnitude and direction of these covariate effects were inconsistent among colonization probabilities (Table 5). Previously unoccupied sites were approximately three times more likely to be colonized if the prior habitat state was dry, compared to sites that remained wet (e.g., ${\hat {\gamma }}_{1}^{[S,S]}=0.11$, SE = 0.06; ${\hat {\gamma }}_{1}^{[U,S]}=0.34$, SE = 0.06; Table 6). Extinction probabilities among sites that remained wet declined by approximately two-thirds for 2015 compared to the prior year (Table 6).

**Table 6:** Model-averaged estimates of initial occupancy (*ψ*), colonization (*γ*), and extinction (ε) parameters, and their standard errors, averaged across all sites.

Parameter	Mean	SE
*ψ*^[*S*]^	0.40	0.40
${\gamma }_{1}^{[S,S]}$	0.09	0.03
${\gamma }_{2}^{[S,S]}$	0.10	0.05
${\gamma }_{1}^{[U,S]}$	0.36	0.09
${\gamma }_{2}^{[U,S]}$	0.36	0.13
${\varepsilon }_{1}^{[S,S]}$	0.30	0.09
${\varepsilon }_{2}^{[S,S]}$	0.08	0.06

Odds and odds ratios are a convenient way to interpret the effects of covariates in occupancy models that utilize logistic regression (MacKenzie et al., 2006). Odds ratios based on the top two models did not differ in terms of the ranks of their magnitude (high values and low values were consistent among models), indicating that covariate effects were consistent among models. We therefore present results based on the model-averaged estimates. Sites with average WD and TS, but with MH were less likely to be occupied than unoccupied during 2013, with odds of 0.76:1 (calculated as *e*^−4.35^ × *e*^4.07×1^). Similarly, sites 1 standard deviation (SD) below average WD (SD = 7.5 cm) without MH were also less likely to be occupied (odds = 0.15:1). Sites 1 SD below average TS (1.62 °C), but with mean WD and no MH were slightly more likely to be occupied than unoccupied (odds = 1.37:1). However, when sites had both MH and low TS (1 SD below average), the overall odds of occupancy vastly improved at 80:1. Even sites with moderate, negative deviations from average covariate values and with MH dramatically improved the odds of occupancy (e.g.,−0.5 SD = 26:1 odds in favor of occupancy).

A full list of model selection results for occupancy analyses is provided in Supplemental Information 3.

## Discussion

From our analyses of individual movement, population demographics, and metapopulation dynamics, we show that *E. tonkawae* is not limited to springs, but can occupy a wide range of headwater stream habitats. This is in contrast to the previous view of habitat for this and other surface dwelling central Texas *Eurycea* as being restricted to habitats within close proximity to springs, and dramatically expands the area *E. tonkawae* may occupy compared to their relatively limited critical habitat designations.

Movement rates, distances individuals traveled, and the presence of recaptured individuals in all of our study sections demonstrated the importance of stream habitat within the spring-influenced stream reach. Movement rates appear to be higher for *E. tonkawae* compared to those reported in other predominantly stream-dwelling salamanders, although differences in study designs and the length of study periods make direct comparisons difficult. For example, in the closely-related *E. naufragia*, movement rates were reported as 1–2% per month into adjacent 5 m sections over a two-year period (Pierce, McEntire & Wall, 2014). Only 18% of *Gyrinophilus porphyriticus* individuals recaptured (*N* = 21∕118) moved beyond 1 m of their original capture site during a two-year period (Lowe, 2003). Almost half of *Psuedotriton ruber* larvae moved >5 m between captures during a one-year study (Cecala, Price & Dorcas, 2009). In comparison, we estimated the probability of movement per two-week period between our 15 m sections as 0.15 (SE = 0.07) for an average sized individual, with larger individuals being more likely to move than smaller ones (Fig. 1). Our approach factors in detection error, whereas a naïve estimate using only a comparison of the proportion of recaptures that moved would be negatively biased. Movement distances observed for *E. tonkawae* (up to 80 m over a four-month period, and one observation of 500 m) were higher than those reported for other *Eurycea* as well. In an eight-month study of *E. bislineata* (*N* = 20), individuals moved a maximum of 30.5 m (Ashton & Ashton, 1978), while *E. naufragia* moved up to 25 m or 50 m (the extents of their spring runs) during a two-year period (Pierce, 2012). Whether movements we observed represented dispersal events or occurred within their home range is uncertain. However, we consider these observations to be an important first step in understanding the movement ecology of this species, a potentially important aspect of amphibian conservation biology (Pittman, Osbourn & Semlitsch, 2014). Furthermore, our results demonstrate the potential of individuals to move the entire linear stream distance of their surface critical habitat, and in at least one case, exceed that distance by over six-fold.

While movement was frequent throughout the stream channel, overall abundances were highest near the spring outlet. This is consistent with previous observations for this and similar species (Tupa & Davis, 1976; Sweet, 1982; Bowles, Sanders & Hansen, 2006; Pierce et al., 2010; Bendik et al., 2014; Diaz et al., 2015). However, the largest proportion of small juveniles was found 80 m downstream, at the limits of our study area and the subsequently updated critical habitat buffer (US Fish and Wildlife Service, 2013). It is unclear whether this demographic pattern is due to dispersal from their natal site, as eggs were not observed during our study (and are rarely observed in the wild; N Bendik, pers. obs., 2015). Juveniles may actively avoid adults to escape predation or drift downstream in the current after hatching from their natal sites, as in other aquatic salamander larvae (Stoneburner, 1978; Bruce, 1986; Fenolio et al., 2014). We did not document movement of any small juveniles (<25 mm total length), although we did find a weak but positive effect of body length on movement probability (Fig. 1). If juvenile drift caused the observed pattern of abundance, it either occurred prior to our study, or at a lower frequency and/or distance than our study design could detect. Individuals may later move upstream to compensate for downstream drift, and some species exhibit upstream-biased movements (Lowe, 2003; Cecala, Price & Dorcas, 2009; Grant et al., 2010). Regardless of the process that produced this pattern, high juvenile abundance well downstream of the large spring outlet indicates the potential importance of these areas for early life-stages of *E. tonkawae*, and we believe that these habitats may extend past the arbitrary 80 m buffer delineated as critical habitat.

Expanding our investigation from a single population to a multi-year study of metapopulation dynamics among headwater streams, we found that initial occupancy was strongly associated with the presence of maidenhair ferns, low temperature variation and shallow water depths. Sites with maidenhair ferns and low temperature variation likely indicate areas where springs or subsurface stream conduits emerge from within the stream channel, acting as connections between surface and subterranean habitat. Models where site occupancy was dependent upon the prior occupancy state were highly supported (compared to a random pattern of site occupancy), indicating some site fidelity of *E. tonkawae* for these habitat types. Prior habitat availability was also important for predicting occupancy dynamics. Previously unoccupied sites were more likely to be colonized if the prior habitat state was dry, demonstrating the propensity of *E. tonkawae* to respond to changing surface habitat conditions and disperse to newly available stream habitats. Yet, habitat characteristics of newly colonized sites were not the same as initially occupied sites, as maidenhair fern presence, temperature variation, and water depth were not consistent predictors of colonization. This result may indicate migration to areas away from groundwater influenced sites during wetter periods, and reflect a general pattern of dynamic occupancy in headwater streams. Shifts in stream occupancy from year to year were also indicated by declines in extinction rates by approximately two-thirds from 2014 to 2015, while colonization rates remained relatively consistent. Colonization of previously dry sites could be occurring from nearby subterranean refugia (e.g., Bendik & Gluesenkamp, 2013), indicating more extensive subterranean habitat beyond documented spring localities (Fig. 3). This is also consistent with our observations of occupancy in thermally stable habitats, as well as results from dye trace studies showing extensive subsurface stream conduits in these headwater streams (Hatch & Johns, 2015). Alternatively, colonization may be occurring via surface movements, as we have demonstrated that individuals are capable of traveling long distances. Collectively, these dynamic occupancy patterns suggest extensive use of stream channels by *E. tonkawae* and their ability to colonize intermittent habitats.

**Figure 4:**
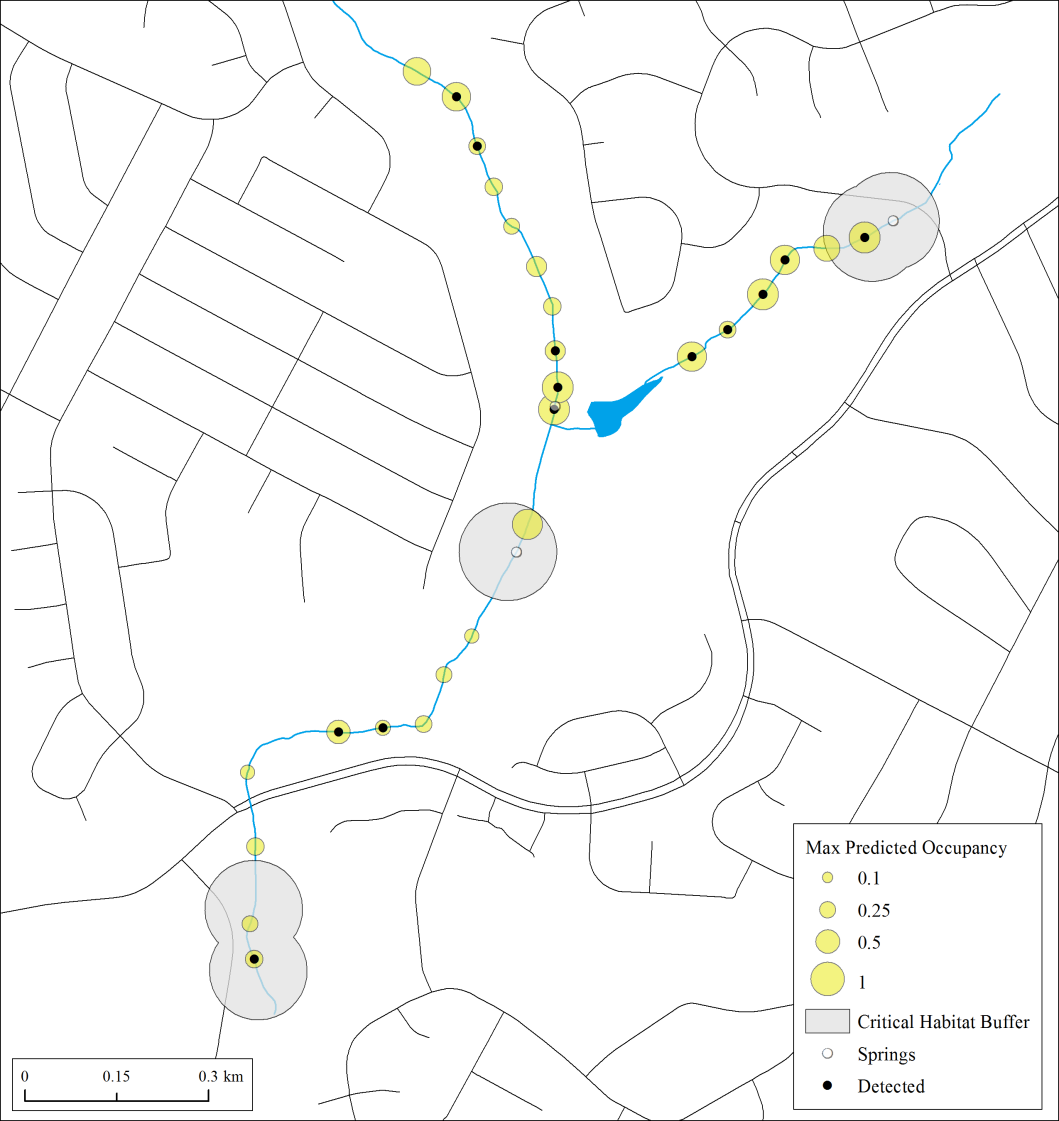
Maximum estimates of predicted occupancy for *E. tonkawae* from 2013–2015 in Bull Creek, Tributary 4, Travis County, Texas. Surface critical habitat is only shown for localities known prior to this study. Predicted values were calculated from initial habitat and occupancy states and their transition probabilities. Missing covariate values were replaced with tributary-specific means.

Results of both naïve and predicted occupancy highlight the extent to which * E. tonkawae* can occur beyond springs and previously documented localities. This is particularly evident in Trib 4, which had never been systematically surveyed prior to our study (Fig. 4). Thus, critical habitat based only on known localities in poorly searched areas may vastly underestimate species presence.

While many studies have demonstrated negative effects of urbanization on stream salamander ecology (for review, see Barrett & Price, 2014), including urbanization-associated declines in *E. tonkawae* abundance (Bendik et al., 2014), we did not find a negative effect on initial occupancy. Rather, we found tributary-specific differences in initial occupancy that did not correspond to their urbanization status. This may be due to sampling error from the small number of tributaries we examined for this effect. However, we did find urban streams were more likely to contain wet sites than those in preserves, which may benefit aquatic salamanders, particularly during dry years. Greater wetted habitat availability within urban streams in Austin is likely due to artificial recharge of the local aquifer systems from urban leakage (Christian, Banner & Mack, 2011). Artificially enhanced or not, wetter tributaries did not correspond to higher colonization rates. In contrast, previously dry sites (which were more common in non-urban catchments) were approximately three times more likely to be colonized compared to wet sites. Colonization in drier tributaries may indicate dispersal events in response to low-water induced conditions such as higher population density and its corresponding effects (i.e., density-dependent dispersal; Matthysen, 2005), whereas in tributaries that remain wet, metapopulation dynamics may be more stable. Higher dry site colonization may also be due to slower predator colonization in previously dry areas, where habitat dynamics result in a period of predator-free space (e.g., Miller et al., 2012). Although we did not directly model predator associations or their occurrence, the negative relationship between water depth and initial occupancy may be due to salamanders avoiding habitat more suitable to predatory fish. An a posteriori Spearman rank correlation test of fish presence and water depth indicates a positive association between these variables (*ρ* = 0.62, *P* = 4.2 × 10^−11^). Paedomorphic *Eurycea* exhibit avoidance behaviors in response to chemical cues from fish (Epp & Gabor, 2008), and individuals may even learn to avoid types of habitat where they have previously encountered predators (Mathis & Unger, 2012). While not the focus of this study, we suggest that further examination of trade-offs between habitat stability, predator occurrence, and other changes to streams associated with urbanization may yield useful insights for management and conservation.

Maintaining population connectivity is of fundamental importance for recovery of amphibian populations (Semlitsch, 2002), and lack of critical habitat protections for stream reaches may increase the likelihood of habitat fragmentation. While land-use changes at the catchment level have the potential to drastically alter stream ecology (Morgan & Cushman, 2005; Walsh et al., 2005) and karst aquifer water quality (Musgrove et al., 2014), habitat continuity within streams is frequently disrupted by culverts, impoundments, utility infrastructure, channelization and other anthropogenic disturbances in urban areas. Even loss of riparian canopy cover can restrict stream-dwelling salamander movement (Cecala, Lowe & Maerz, 2014). Stream modifications (e.g., those requiring a Clean Water Act, Section 404 permit; 404, 33 U.S.C. §1344) are most likely to trigger the type of legal protections that are afforded by critical habitat designation through the interagency cooperation provision of the Endangered Species Act of 1973 (16 U.S.C. §1536(a)(2)). Although some might argue that federal administrative cooperation is the only protection afforded by critical habitat in the United States, these designations and their accompanying assumptions about the distribution and habitat of species may influence other conservation policy decisions as well. One example of this is the 4(d) rule for *E. naufragia*, which establishes specific conditions where take of this species is permitted. In essence, the rule is a list of guidelines for developers to follow in lieu of requiring an incidental take permit or Habitat Conservation Plan (US Fish and Wildlife Service, 2015b). The 4(d) rule was originally developed as an ordinance by the City of Georgetown and based on (yet-to-be finalized) critical habitat, and allows for disturbance of stream habitats outside 50 m of known localities. If *E. naufragia* use stream habitats similarly to *E. tonkawae*, stream disturbances as allowed by this 4(d) rule could potentially lead to loss of stream habitats and fragmentation among populations. Conservation actions that target species recovery may fall short when critical habitat fails to protect metapopulation connectivity or accurately represent species’ habitat use.

## Conclusions

Based on our results indicating extensive use of stream habitats by *E. tonkawae*, we recommend headwater streams be included as critical habitat for *E*. *tonkawae* and carefully considered for similar species either awaiting final critical habitat or listing determinations. Beyond its regulatory function, critical habitat should represent an acknowledgement of the habitat necessary for species recovery, rather than a smattering of disjunct areas based on occurrence records as is currently the case for *E. tonkawae* and related species. Unfortunately, consideration of only known localities is common practice when designating critical habitat, despite the potential importance of unoccupied habitats for colonization or translocations to support recovery efforts (Camaclang et al., 2015). Known localities likely only represent a fraction of occupied areas for species that occur in subterranean and intermittent habitats, such as central Texas *Eurycea*, further underscoring the need to test assumptions of habitat use. As others have argued (Hoekstra, Fagan & Bradley, 2002), we believe critical habitat should be designated biologically according to species habitat requirements, not just by historically-occupied sites, and that habitat models should be used to guide these designations. Estimates from species-habitat models may be used to predict occupancy dynamics under varying habitat/climate conditions, in other headwater streams, or for similar species. These predictions may provide guidance for conservation actions such as habitat restoration and site selection for species reintroductions as part of an adaptive management strategy. The success of recovery, however, is dependent upon the accurate recognition and protection of the full range of habitats necessary for species survival.

## Supplemental Information

10.7717/peerj.1817/supp-1 Supplemental Information 1 Descriptive analysis of environmental variables Tables S1-S8 & Figures S1-S4

10.7717/peerj.1817/supp-2 Supplemental Information 2 Results from capture-recapture model selection and model averaged parameter estimates

10.7717/peerj.1817/supp-3 Supplemental Information 3 Occupancy model selection results

10.7717/peerj.1817/supp-4 Supplemental Information 4 Open population dataset for *N* estimation

10.7717/peerj.1817/supp-5 Supplemental Information 5 Dataset for multistrata analysis

10.7717/peerj.1817/supp-6 Supplemental Information 6 Occupancy dataset

## Additional Information and Declarations

### Field Study Permissions

The following information was supplied relating to field study approvals (i.e., approving body and any reference numbers): We performed all studies under the authority of Texas Parks & Wildlife scientific permit SPR-0113-006 and Federal Fish and Wildlife permit TE833851.

### Data Availability

The following information was supplied regarding data availability: Data used for capture-recapture and occupancy analyses are available in Supplemental Information. The full suite of occupancy data is available at the following URL: https://data.austintexas.gov/d/tzxa-xaix?category=Environmentalview_name=Jollyville-Salamander-Occupancy-Project-Data.
