# Supplementary material for: Movement, demographics, and occupancy dynamics of a federally-threatened salamander: evaluating the adequacy of critical habitat
Source: PeerJ. 2016 Mar 15;4:e1817. doi: 10.7717/peerj.1817 (PMC4797769; doi:10.7717/peerj.1817)
Supplement: Supplemental Information 1 — Tables S1-S8 & Figures S1-S4 [file peerj-04-1817-s001.pdf]

Table S1. Condition index analysis to assess multicollinearity among potential continuous predictor variables for occupancy analysis.

Year: 2013			Eigenvectors				
Number	Eigenvalue	Condition Index	DO	Rock Cover	Depth	Temp	Temp_SD
1	2.18E+05	1	-0.123943	-0.9601476	-0.00594	-0.24875	-0.02903
2	8.51E+03	5.057749	-0.459649	0.27925766	-0.02335	-0.83521	-0.1123
3	3.07E+02	26.614959	**0.635348**	0.01057917	-0.01922	**-0.43163**	**0.63996**
4	8.28E+01	51.285973	**-0.60785**	-0.0043092	0.022739	**0.230317**	**0.759564**
5	4.10E+00	230.387119	-0.014574	-0.0011244	-0.99927	0.034541	0.007772
Year: 2014			Eigenvectors				
Number	Eigenvalue	Condition Index	DO	Rock Cover	Depth	Temp	Temp_SD
1	2.14E+05	1	-0.140246	-0.9481085	-0.00791	-0.28376	-0.02898
2	1.18E+04	4.245746	-0.465284	0.31747997	-0.02452	-0.8205	-0.09433
3	2.10E+02	31.892887	**0.853793**	0.01722644	0.017034	**-0.49599**	**0.156361**
4	1.14E+02	43.355256	**-0.185584**	-0.0002284	0.033242	-0.00874	**0.982027**
5	5.13E+00	203.962791	2.09E-02	3.56E-06	-0.999	1.36E-02	3.79E-02
Year: 2015			Eigenvectors				
Number	Eigenvalue	Condition Index	DO	Rock Cover	Depth	Temp	Temp_SD
1	2.81E+05	1	-0.136966	-0.9419887	-0.00799	-0.30556	-0.02162
2	1.85E+04	3.893701	-0.401913	0.33555692	-0.02707	-0.85014	-0.04891
3	2.46E+02	33.7824	**0.888523**	0.00562203	-0.00316	**-0.42734**	**0.166928**
4	4.77E+01	76.815656	**-0.173635**	-0.0049698	0.000181	0.023484	**0.984517**
5	6.68E+00	205.168903	0.009136	-0.0015731	-0.9996	0.02682	0.001147

Note: Values in bold are eigenvectors judged to contribute substantial collinearity for condition indices > 30.

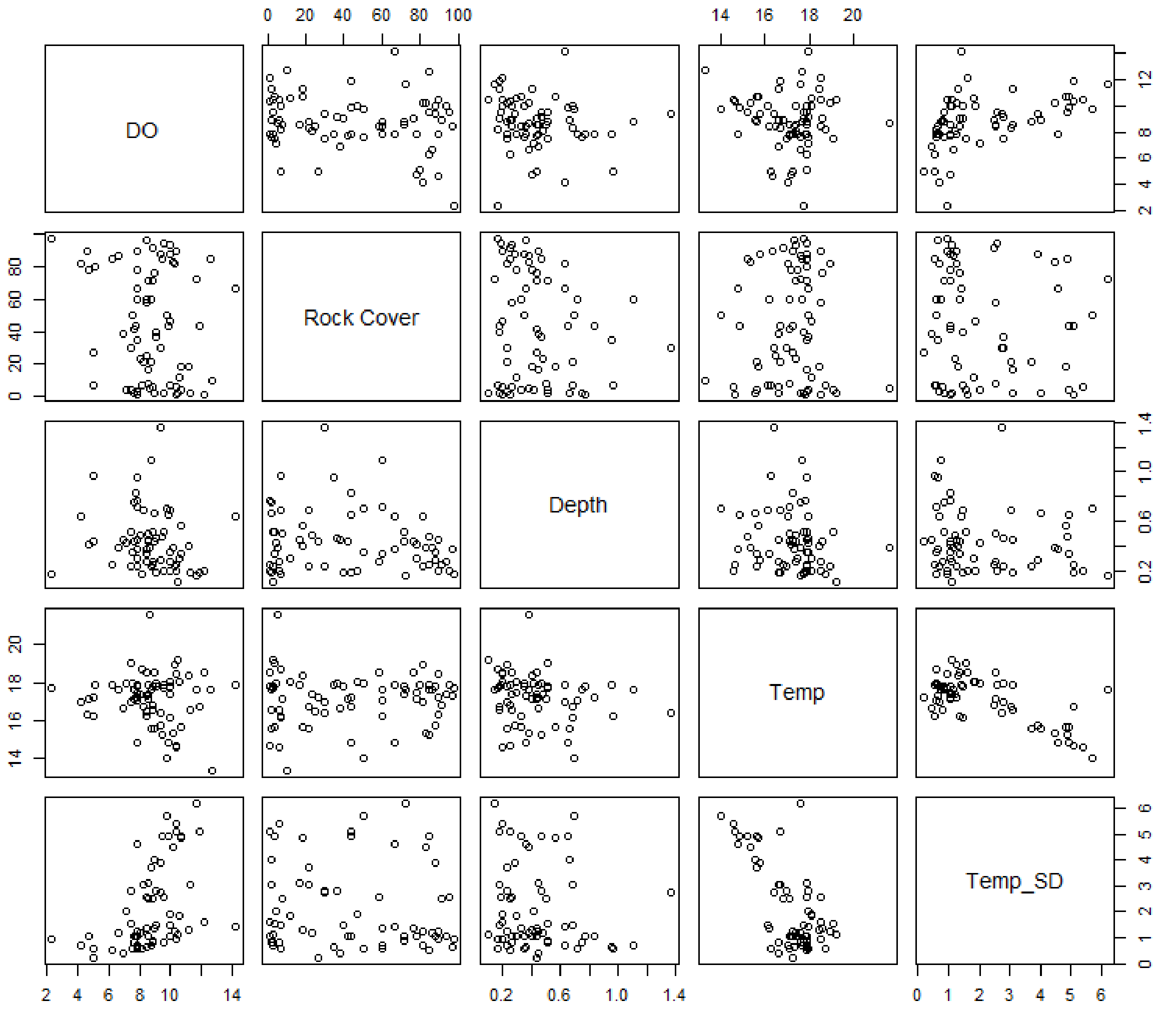

Figure S1. Scatterplots among potential continuous predictor variables for occupancy analysis, year 2013.

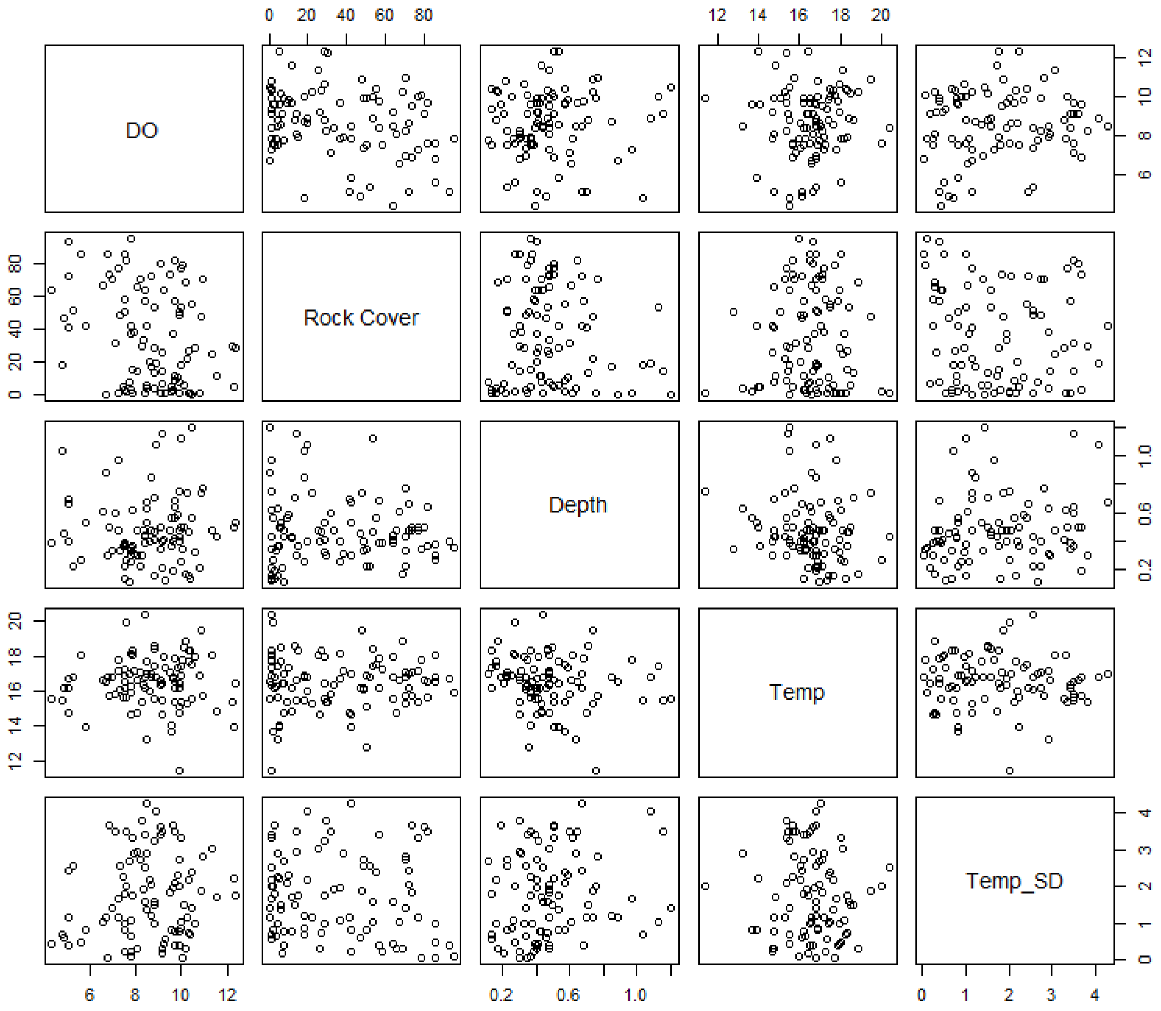

Figure S2. Scatterplots among potential continuous predictor variables for occupancy analysis, year 2014.

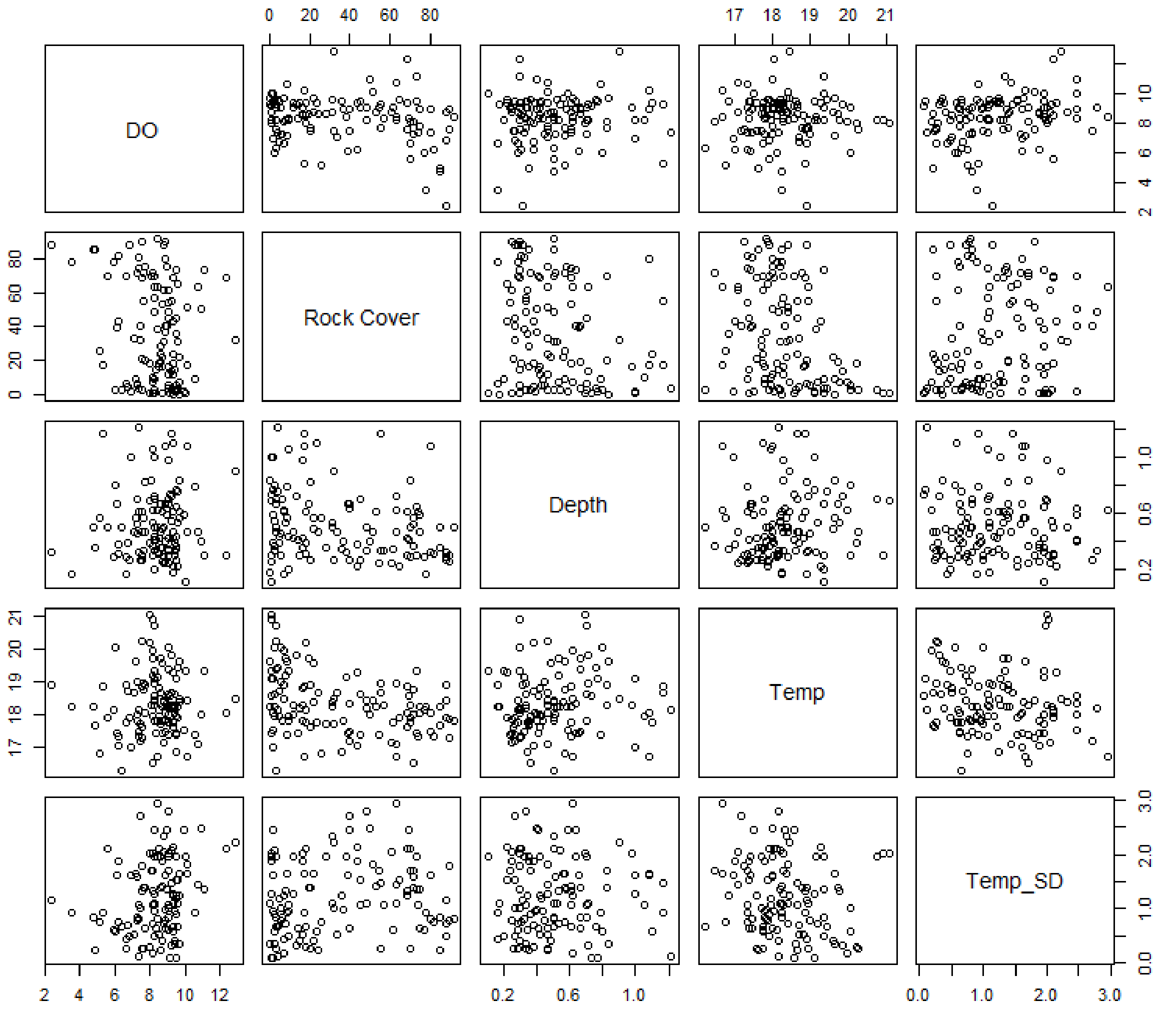

Figure S3. Scatterplots among potential continuous predictor variables for occupancy analysis, year 2015.

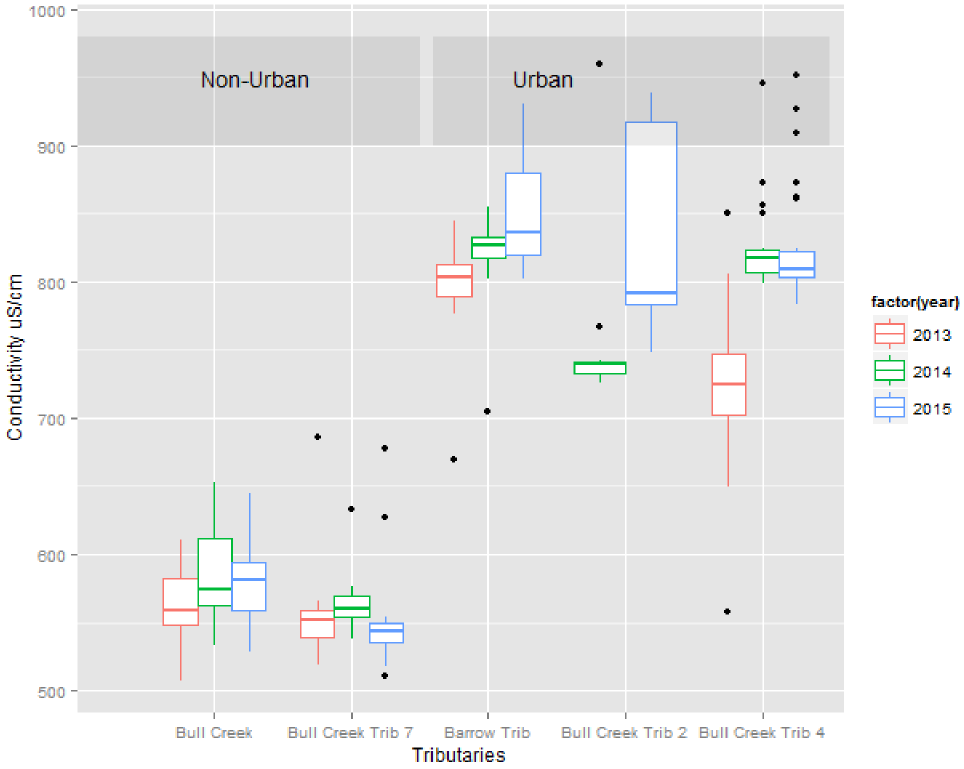

Figure S4. Specific conductance (μS/cm) variation among sites by tributary and year.

Table S2. Chi-squared test results for categorical variables.

Variable	TRIB χ^2 statistic (*P*)	URB χ^2 statistic (*P*)
Calcium Carbonate Deposition	13.975 (0.00737)	6.554 (0.0105)
Maidenhair Fern	27.253 (1.77e-05)	20.856 (4.95e-06)

Table S3. One-way ANOVA for differences by TRIB, year 2013.

	Dependent variable:		
	Depth	Rock Cover	Temp. Variation
	(1)	(2)	(3)
Bull Creek	−0.011	−9.360	-2.869^{***}
	(0.098)	(11.106)	(0.414)
Bull Creek Trib 4	0.069	3.402	-3.081^{***}
	(0.078)	(9.938)	(0.329)
Bull Creek Trib 7	0.131	-33.289^{***}	-1.725^{***}
	(0.086)	(10.920)	(0.368)
Constant	0.381^{***}	52.716^{***}	4.055^{***}
	(0.060)	(7.604)	(0.252)
Observations	67	72	66
R^2	0.048	0.178	0.610
Adjusted R^2	0.003	0.142	0.591
Residual Std. Error	0.246 (df = 63)	31.350 (df = 68)	1.038 (df = 62)
F Statistic	1.065 (df = 3; 63)	4.906^{***} (df = 3; 68)	32.372^{***} (df = 3; 62)

Note: *p<0.1; **p<0.05; ***p<0.01

Table S4. One-way ANOVA for differences by URB, year 2013.

	Dependent variable:		
	Depth	Rock Cover	Temp. Variation
	(1)	(2)	(3)
Urbanized	−0.036	23.702^{***}	0.379
	(0.062)	(7.600)	(0.412)
Constant	0.458^{***}	31.005^{***}	1.873^{***}
	(0.048)	(5.735)	(0.325)
Observations	67	72	66
R^2	0.005	0.122	0.013
Adjusted R^2	−0.010	0.109	−0.002
Residual Std. Error	0.247 (df = 65)	31.933 (df = 70)	1.625 (df = 64)
F Statistic	0.335 (df = 1; 65)	9.725^{***} (df = 1; 70)	0.842 (df = 1; 64)

Note: *p<0.1; **p<0.05; ***p<0.01

Table S5. One-way ANOVA for differences by TRIB, year 2014.

	Dependent variable:		
	Depth (1)	Rock Cover (2)	Temp. Variation (3)
Bull Creek	0.008	−1.315	−1.859***
	(0.088)	(8.205)	(0.271)
Bull Creek Trib 2	−0.003	21.407**	0.207
	(0.100)	(9.661)	(0.329)
Bull Creek Trib 4	−0.035	7.352	−1.463***
	(0.090)	(8.436)	(0.276)
Bull Creek Trib 7	0.040	−20.156**	−1.353***
	(0.092)	(8.730)	(0.292)
Constant	0.471***	35.542***	2.849***
	(0.073)	(6.256)	(0.210)
Observations	90	97	90
R^2	0.011	0.187	0.483
Adjusted R^2	−0.035	0.152	0.459
Residual Std. Error	0.243 (df = 85)	26.542 (df = 92)	0.839 (df = 85)
F Statistic	0.245 (df = 4; 85)	5.289*** (df = 4; 92)	19.851*** (df = 4; 85)

Note: *p<0.1; **p<0.05; ***p<0.01

Table S6. One-way ANOVA for differences by URB, year 2014.

	Dependent variable:		
	Depth (1)	Rock Cover (2)	Temp. Variation (3)
Urbanized	−0.039	17.753***	1.039***
	(0.051)	(5.620)	(0.216)
Constant	0.492***	26.091***	1.200***
	(0.036)	(4.154)	(0.159)
Observations	90	97	90
R^2	0.007	0.095	0.208
Adjusted R^2	−0.004	0.086	0.199
Residual Std. Error	0.240 (df = 88)	27.556 (df = 95)	1.021 (df = 88)
F Statistic	0.605 (df = 1; 88)	9.979*** (df = 1; 95)	23.124*** (df = 1; 88)

Note: *p<0.1; **p<0.05; ***p<0.01

Table S7. One-way ANOVA for differences by TRIB, year 2015.

	Dependent variable:		
	Depth (1)	Rock Cover (2)	Temp. Variation (3)
Bull Creek	0.540^{**}	-7.705	0.546^{***}
	(0.232)	(7.579)	(0.165)
Bull Creek Trib 2	0.179	24.702^{***}	0.392^{**}
	(0.274)	(8.930)	(0.194)
Bull Creek Trib 4	-0.430^{*}	3.857	0.685^{***}
	(0.243)	(7.943)	(0.173)
Bull Creek Trib 7	1.025^{***}	-23.405^{***}	-0.349^{**}
	(0.248)	(8.081)	(0.176)
Constant	18.030^{***}	38.246^{***}	0.929^{***}
	(0.185)	(6.038)	(0.131)
Observations	118	118	118
R^2	0.292	0.238	0.324
Adjusted R^2	0.267	0.211	0.300
Residual Std. Error (df = 113)	0.806	26.317	0.573
F Statistic (df = 4; 113)	11.661^{***}	8.803^{***}	13.561^{***}
Note:			$^{*}p<0.1$; $^{**}p<0.05$; $^{***}p<0.01$

Table S8. One-way ANOVA for differences by URB, year 2015.

	Dependent variable:		
	Depth (1)	Rock Cover (2)	Temp. Variation (3)
Urbanized	-0.031	22.439^{***}	0.225^{*}
	(0.045)	(5.069)	(0.125)
Constant	0.530^{***}	23.930^{***}	1.098^{***}
	(0.033)	(3.644)	(0.090)
Observations	118	118	118
R^2	0.004	0.145	0.027
Adjusted R^2	-0.005	0.137	0.019
Residual Std. Error (df = 116)	0.246	27.514	0.678
F Statistic (df = 1; 116)	0.459	19.599^{***}	3.249^{*}
Note:			$^{*}p<0.1$; $^{**}p<0.05$; $^{***}p<0.01$
