# Supplementary material for: Movement, demographics, and occupancy dynamics of a federally-threatened salamander: evaluating the adequacy of critical habitat
Source: PeerJ. 2016 Mar 15;4:e1817. doi: 10.7717/peerj.1817 (PMC4797769; doi:10.7717/peerj.1817)
Supplement: Supplemental Information 2 [file peerj-04-1817-s002.pdf]

Table S9. Multi-state capture-recapture model selection results. φ = apparent survival; p = detection probability; ψ = transition probability; BL = body length covariate; t = time variation; (.) = constant.

Model	AICc	ΔAICc	w_i	Model Likelihood	K	Deviance
φ(BL) $p(t)$ ψ (BL)	767.2314	0	0.4125	1	11	744.4771
φ(BL) $p(t$ + BL) ψ(BL)	768.7686	1.5372	0.19126	0.4637	12	743.8746
φ(BL) $p(t)$ ψ(.)	769.2978	2.0664	0.1468	0.3559	10	748.671
φ(BL) $p(t$ + BL) ψ(.)	770.8228	3.5914	0.06848	0.166	11	748.0685
$\varphi(t$ + BL) $p(t)$ ψ(BL)	771.8204	4.589	0.04159	0.1008	16	738.2435
φ(.) $p(t$ + BL) ψ(BL)	772.7178	5.4864	0.02655	0.0644	11	749.9635
$\varphi(t)$ p(BL) ψ(BL)	773.0411	5.8097	0.02259	0.0548	11	750.2868
$\varphi(t$ + BL) $p(t$ + BL) ψ(BL)	773.4472	6.2158	0.01844	0.0447	17	737.6682
$\varphi(t$ + BL) $p(t)$ ψ(.)	773.8247	6.5933	0.01527	0.037	15	742.4374
$\varphi(t$ + BL) p(.) ψ(BL)	774.5002	7.2688	0.01089	0.0264	11	751.7459
φ(.) $p(t$ + BL) ψ(.)	774.7842	7.5528	0.00945	0.0229	10	754.1574
$\varphi(t$ + BL) p(BL) ψ(BL)	775.024	7.7926	0.00838	0.0203	12	750.13
$\varphi(t)$ p(BL) ψ(.)	775.1075	7.8761	0.00804	0.0195	10	754.4807
$\varphi(t$ + BL) $p(t$ + BL) ψ(.)	775.4388	8.2074	0.00681	0.0165	16	741.862
$\varphi(t$ + BL) p(.) ψ(.)	776.5665	9.3351	0.00388	0.0094	10	755.9397
$\varphi(t$ + BL) p(BL) ψ(.)	777.0782	9.8468	0.003	0.0073	11	754.3239
$\varphi(t)$ $p(t$ + BL) ψ(BL)	777.1462	9.9148	0.0029	0.007	16	743.5694
$\varphi(t)$ $p(t$ + BL) ψ(.)	779.1505	11.9191	0.00106	0.0026	15	747.7633
φ(.) $p(t)$ ψ(BL)	779.9301	12.6987	0.00072	0.0017	10	759.3033
φ(BL) p(.) ψ(BL)	780.9248	13.6934	0.00044	0.0011	5	770.7563
φ(.) $p(t)$ ψ(.)	782.0085	14.7771	0.00026	0.0006	9	763.4972
φ(BL) p(BL) ψ(BL)	782.9328	15.7014	0.00016	0.0004	6	770.6962
φ(BL) p(.) ψ(.)	783.0622	15.8308	0.00015	0.0004	4	774.9502
$\varphi(t)$ p(.) ψ(BL)	783.2237	15.9923	0.00014	0.0003	10	762.5969
φ(.) p(BL) ψ(BL)	784.2763	17.0449	0.00008	0.0002	5	774.1078
φ(BL) p(BL) ψ(.)	785.0586	17.8272	0.00006	0.0001	5	774.8901
$\varphi(t)$ p(.) ψ(.)	785.3021	18.0707	0.00005	0.0001	9	766.7908
$\varphi(t)$ $p(t)$ ψ(BL)	786.0548	18.8234	0.00003	0.0001	14	756.8444
φ(.) p(BL) ψ(.)	786.4137	19.1823	0.00003	0.0001	4	778.3017
$\varphi(t)$ $p(t)$ ψ(.)	790.2486	23.0172	0	0	14	761.0383
φ(.) $p(t)$ $\psi(t)$	792.8393	25.6079	0	0	15	761.452
φ(.) p(.) ψ(BL)	793.8786	26.6472	0	0	4	785.7666
φ(.) p(.) ψ(.)	796.0275	28.7961	0	0	3	789.9605
$\varphi(t)$ p(.) $\psi(t)$	796.1329	28.9015	0	0	15	764.7456
$\varphi(t)$ $p(t)$ $\psi(t)$	803.7107	36.4793	0	0	21	758.9931
φ(.) p(.) $\psi(t)$	806.4267	39.1953	0	0	9	787.9153

Table S10. POPAN capture-recapture model selection results. φ = apparent survival; p = detection probability; *PENT* = probability of entrance; N = superpopulation size; BL = body length covariate; t = time variation; (.) = constant.

Model	*QAICc*	*Δ*QAICc	w_i	Model Likelihood	*K*	QDeviance
φ(BL) p(.) *PENT*(t) N(section)	599.4443	0	0.2347	1	18	561.5443
φ(t) p(BL) *PENT*(t) N(section)	600.218	0.7737	0.15941	0.6792	24	548.8282
φ(.) p(BL) *PENT*(t) N(section)	600.6872	1.2429	0.12607	0.5371	18	562.7872
φ(t + BL) p(.) *PENT*(t) N(section)	600.943	1.4987	0.11094	0.4727	24	549.5532
φ(BL) p(t) *PENT*(t) N(section)	601.4296	1.9853	0.08698	0.3706	25	547.7469
φ(BL) p(BL) *PENT*(t) N(section)	601.5557	2.1114	0.08166	0.3479	19	561.4387
φ(.) p(t+BL) *PENT*(t) N(section)	601.7806	2.3363	0.07298	0.3109	24	550.3908
φ(t + BL) p(BL) *PENT*(t) N(section)	602.3918	2.9475	0.05376	0.2291	25	548.709
φ(BL) p(t+BL) *PENT*(t) N(section)	603.4895	4.0452	0.03105	0.1323	26	547.5008
φ(t + BL) p(t) *PENT*(t) N(section)	605.5135	6.0692	0.01129	0.0481	28	544.8735
φ(.) p(t) *PENT*(t) N(section)	605.8296	6.3853	0.00964	0.0411	24	554.4398
φ(.) p(.) *PENT*(t) N(section)	606.2599	6.8156	0.00777	0.0331	18	568.3598
φ(t) p(.) *PENT*(t) N(section)	606.883	7.4387	0.00569	0.0242	24	555.4932
φ(t + BL) p(t+BL) *PENT*(t) N(section)	607.3819	7.9376	0.00443	0.0189	29	544.3963
φ(t) p(t+BL) *PENT*(t) N(section)	607.9528	8.5085	0.00333	0.0142	29	544.9671
φ(t) p(t) *PENT*(t) N(section)	612.9022	13.4579	0.00028	0.0012	28	552.2622

Table S11. Multi-state capture-recapture model averaged parameter results, assuming mean covariate value (BL = 24.8).

Parameter	Estimate	SE	LCL	UCL
ψ	0.07346	0.035942	0.027392	0.182471
p_1	0.326426	0.109551	0.154339	0.562712
p_2	0.420706	0.093127	0.255626	0.605653
p_3	0.553942	0.099019	0.361504	0.731464
p_4	0.302526	0.073368	0.179931	0.461629
p_5	0.412741	0.095612	0.244939	0.603603
p_6	0.38042	0.093248	0.220427	0.571421
p_7	0.108536	0.076629	0.025135	0.365052
φ_1	0.642827	0.097378	0.439394	0.805171
φ_2	0.614587	0.071187	0.469437	0.741863
φ_3	0.643786	0.084916	0.466569	0.788782
φ_4	0.637109	0.108042	0.412634	0.814385
φ_5	0.612164	0.09533	0.418102	0.776156
φ_6	0.659599	0.120892	0.402801	0.847719
φ_7	0.629006	0.164794	0.298139	0.871253

Table S12. POPAN capture-recapture model averaged parameter results, assuming mean covariate value (BL = 24.8).

Parameter	Estimate	SE	LCL	UCL
p_1	0.526795	0.259426	0.126477	0.895392
p_2	0.37686	0.094102	0.216141	0.57016
p_3	0.392935	0.08246	0.247397	0.560345
p_4	0.429317	0.10668	0.242676	0.638484
p_5	0.374195	0.081577	0.232	0.542033
p_6	0.404068	0.088823	0.247586	0.58284
p_7	0.381771	0.084174	0.234864	0.554031
φ_1	0.581573	0.118614	0.348388	0.783231
φ_2	0.552934	0.08848	0.380127	0.713834
φ_3	0.660563	0.165559	0.314015	0.892163
φ_4	0.543427	0.090015	0.368902	0.707903
φ_5	0.606625	0.120959	0.363467	0.806378
φ_6	0.5706	0.111799	0.35205	0.764703
φ_7	0.441608	0.202849	0.13623	0.798619
