# Supplementary material for: Movement, demographics, and occupancy dynamics of a federally-threatened salamander: evaluating the adequacy of critical habitat
Source: PeerJ. 2016 Mar 15;4:e1817. doi: 10.7717/peerj.1817 (PMC4797769; doi:10.7717/peerj.1817)
Supplement: Supplemental Information 3 [file peerj-04-1817-s003.pdf]

Table S13. Occupancy model selection results. $\psi^{[S]}$ = initial occupancy probability; $\gamma^{[U,S]}$ = probability of colonization if site was unsuitable the previous year; $\gamma^{[S,S]}$ = probability of colonization if site was suitable the previous year; $\varepsilon$ = probability of extinction if site was suitable the previous year; $\pi^{[S]}$ = initial probability of suitable habitat; $\eta^{[S,S]}$ = probability of habitat remaining suitable if site was suitable the previous year. $\eta^{[U,S]}$ = probability of habitat becoming suitable if site was unsuitable the previous year. *p* = probability of species detection. TRIB and URB reflect site groupings by tributary and urbanization category, respectively. Time variation is indicated by *t* while parameters that were modeled as constant across years are indicated by a dot, "(.)". TS = temperature stability; WD = water depth; RC = rock cover; MH = presence of maidenhair ferns; CA = calcium carbonate deposition.

| Model | AICc | ΔAICc | $w_i$ | Model Likelihood | *K* | *-2l* |
|---|---|---|---|---|---|---|
| $\psi^{[S]}\,\gamma^{[U,S]}\,\gamma^{[S,S]*}(\text{TS}_t + \text{WD}_t + \text{MH}), \varepsilon(t), \pi^{[S]}(\text{URB}), \eta^{[S,S]}(t), \eta^{[U,S]}(t), p(\text{TRIB})$ | 753.76 | 0 | 0.51 | 1 | 24 | 703.74 |
| $\psi^{[S]}\,\gamma^{[U,S]}\,\gamma^{[S,S]*}(\text{TS}_t + \text{WD}_t + \text{CA} + \text{MH}), \varepsilon(t), \pi^{[S]}(\text{URB}), \eta^{[S,S]}(t), \eta^{[U,S]}(t), p(\text{TRIB})$ | 753.87 | 0.11 | 0.4827 | 0.947 | 26 | 699.5 |
| $\psi^{[S]}\,\gamma^{[U,S]}\,\gamma^{[S,S]*}(\text{TS}_t + \text{WD}_t), \varepsilon(t), \pi^{[S]}(\text{URB}), \eta^{[S,S]}(t), \eta^{[U,S]}(t), p(\text{TRIB})$ | 762.62 | 8.86 | 0.0061 | 0.0119 | 22 | 716.92 |
| $\psi^{[S]}\,\gamma^{[U,S]}\,\gamma^{[S,S]*}(\text{CA} + \text{TS}_t + \text{WD}_t), \varepsilon(t), \pi^{[S]}(\text{URB}), \eta^{[S,S]}(t), \eta^{[U,S]}(t), p(\text{TRIB})$ | 766.14 | 12.38 | 0.001 | 0.002 | 24 | 716.12 |
| $\psi^{[S]}\,\gamma^{[U,S]}\,\gamma^{[S,S]*}(\text{RC}_t + \text{TS}_t + \text{WD}_t), \varepsilon(t), \pi^{[S]}(\text{URB}), \eta^{[S,S]}(t), \eta^{[U,S]}(t), p(\text{TRIB})$ | 769.74 | 15.98 | 0.0002 | 0.0003 | 25 | 717.55 |
| $\psi^{[S]}\,\gamma^{[U,S]}\,\gamma^{[S,S]*}(\text{TS}_t), \varepsilon(t), \pi^{[S]}(\text{URB}), \eta^{[S,S]}(t), \eta^{[U,S]}(t), p(\text{TRIB})$ | 773.3 | 19.54 | 0 | 0.0001 | 19 | 734.03 |
| $\psi^{[S]}\,\gamma^{[U,S]}\,\gamma^{[S,S]*}(\text{RC}_t), \varepsilon(t), \pi^{[S]}(\text{URB}), \eta^{[S,S]}(t), \eta^{[U,S]}(t), p(\text{TRIB})$ | 777.98 | 24.22 | 0 | 0 | 19 | 738.71 |
| $\psi^{[S]}\,\gamma^{[U,S]}\,\gamma^{[S,S]*}(\text{CA} + \text{MH}), \varepsilon(t), \pi^{[S]}(\text{URB}), \eta^{[S,S]}(t), \eta^{[U,S]}(t), p(\text{TRIB})$ | 778.15 | 24.39 | 0 | 0 | 20 | 736.75 |
| $\psi^{[S]}\,\gamma^{[U,S]}\,\gamma^{[S,S]*}(\text{RC}_t + \text{TS}_t), \varepsilon(t), \pi^{[S]}(\text{URB}), \eta^{[S,S]}(t), \eta^{[U,S]}(t), p(\text{TRIB})$ | 779.38 | 25.62 | 0 | 0 | 22 | 733.68 |
| $\psi^{[S]}\,(\text{TRIB})\,\gamma^{[U,S]}(t), \gamma^{[S,S]}(t), \varepsilon(t), \pi^{[S]}(\text{TRIB}), \eta^{[S,S]}(t), \eta^{[U,S]}(t), p(\text{TRIB})$ | 779.58 | 25.82 | 0 | 0 | 22 | 733.88 |
| $\psi^{[S]}\,\gamma^{[U,S]}\,\gamma^{[S,S]*}(\text{MH}), \varepsilon(t), \pi^{[S]}(\text{URB}), \eta^{[S,S]}(t), \eta^{[U,S]}(t), p(\text{TRIB})$ | 780.64 | 26.88 | 0 | 0 | 18 | 743.5 |
| $\psi^{[S]}\,\gamma^{[U,S]}\,\gamma^{[S,S]*}(\text{CA}), \varepsilon(t), \pi^{[S]}(\text{URB}), \eta^{[S,S]}(t), \eta^{[U,S]}(t), p(\text{TRIB})$ | 783.53 | 29.77 | 0 | 0 | 18 | 746.39 |
| $\psi^{[S]}\,\gamma^{[U,S]}\,\gamma^{[S,S]*}(\text{WD}_t), \varepsilon(t), \pi^{[S]}(\text{URB}), \eta^{[S,S]}(t), \eta^{[U,S]}(t), p(\text{TRIB})$ | 784.89 | 31.13 | 0 | 0 | 19 | 745.62 |
| $\psi^{[S]}\,\gamma^{[U,S]}\,\gamma^{[S,S]*}(\text{RC}_t + \text{WD}_t), \varepsilon(t), \pi^{[S]}(\text{URB}), \eta^{[S,S]}(t), \eta^{[U,S]}(t), p(\text{TRIB})$ | 785.45 | 31.69 | 0 | 0 | 22 | 739.75 |
| $\psi^{[S]}\,(\text{URB})\,\gamma^{[U,S]}(t), \gamma^{[S,S]}(t), \varepsilon(t), \pi^{[S]}(\text{URB}), \eta^{[S,S]}(t), \eta^{[U,S]}(t), p(\text{TRIB})$ | 785.88 | 32.12 | 0 | 0 | 19 | 746.61 |
| $\psi^{[S]}, \gamma^{[U,S]}(t), \gamma^{[S,S]}(t), \varepsilon(t), \pi^{[S]}(\text{URB}), \eta^{[S,S]}(t), \eta^{[U,S]}(t), p(\text{TRIB})$ | 787.87 | 34.11 | 0 | 0 | 18 | 750.73 |
| $\psi^{[S]}, \gamma^{[U,S]}(t), \gamma^{[S,S]}(t), \varepsilon(t), \pi^{[S]}(\text{TRIB}), \eta^{[S,S]}(t), \eta^{[U,S]}(t), p(\text{TRIB})$ | 792.7 | 38.94 | 0 | 0 | 21 | 749.15 |
| $\psi^{[S]}, \gamma^{[U,S]}{=}\gamma^{[S,S]}(t), \varepsilon(t), \pi^{[S]}(\text{URB}), \eta^{[S,S]}(t), \eta^{[U,S]}(t), p(\text{TRIB})$ | 794.58 | 40.82 | 0 | 0 | 16 | 761.68 |
| $\psi^{[S]}, \gamma^{[U,S]}(t), \gamma^{[S,S]}(t), \varepsilon\,(t), \pi^{[S]}(.), \eta^{[S,S]}(t), \eta^{[U,S]}(t), p(\text{TRIB})$ | 794.75 | 40.99 | 0 | 0 | 17 | 759.73 |
| $\psi^{[S]}, \gamma^{[U,S]}(t), \gamma^{[S,S]}(t), \varepsilon\,(t), \pi^{[S]}(.)\ \eta^{[S,S]}(t)\ \eta^{[U,S]}(t)\ p(.)$ | 795.82 | 42.06 | 0 | 0 | 13 | 769.22 |
| $\psi^{[S]}, \gamma^{[U,S]}(t), \gamma^{[S,S]}(t), \varepsilon\,(t), \pi^{[S]}(.), \eta^{[S,S]}(t), \eta^{[U,S]}(t), p(t)$ | 799.44 | 45.68 | 0 | 0 | 15 | 768.65 |
| $\psi^{[S]}, \gamma^{[U,S]}(t), \gamma^{[S,S]}(t), \varepsilon\,(t), \pi^{[S]}(.), \eta^{[S,S]}(t), \eta^{[U,S]}(t), p(\text{TRIB} + t)$ | 800.23 | 46.47 | 0 | 0 | 20 | 758.83 |

Table S13 continued.

| Model | AICc | ΔAICc | $w_i$ | Model Likelihood | $K$ | $-2l$ |
|---|---|---|---|---|---|---|
| $\psi^{[S]}, \gamma^{[U,S]}(t), \gamma^{[S,S]}(t), \varepsilon(t), \pi^{[S]}(.), \eta^{[S,S]}(.)\ \eta^{[U,S]}(.)\ p(\text{TRIB})$ | 828.51 | 74.75 | 0 | 0 | 15 | 797.72 |
| $\psi^{[S]}, \gamma^{[U,S]}(t), \gamma^{[S,S]}(t), \varepsilon(t), \pi^{[S]}(.), \eta^{[S,S]}=\eta^{[U,S]}(t), p(\text{TRIB})$ | 829.32 | 75.56 | 0 | 0 | 15 | 798.53 |
| $\psi^{[S]}, \gamma^{[U,S]}(t), \gamma^{[S,S]}(t), \varepsilon(t), \pi^{[S]}(.), \eta^{[S,S]}=\eta^{[U,S]}(.), p(\text{TRIB})$ | 859.91 | 106.15 | 0 | 0 | 14 | 831.22 |
| $\psi^{[S]}, \gamma^{[U,S]}=\gamma^{[S,S]}=(1-\varepsilon)(t), \pi^{[S]}(\text{URB})\ \eta^{[S,S]}(t)\ \eta^{[U,S]}(t)\ p(\text{TRIB})$ | 1303.23 | 549.47 | 0 | 0 | 14 | 1274.54 |

*Indicates covariates indicated are the same for both initial occupancy and colonization parameters. The effect of covariates on colonization was assumed to be independent of the prior habitat state. Effective sample size was assumed to be 620.
